# No universals in the cultural evolution of kinship terminology

**DOI:** 10.1017/ehs.2020.41

**Published:** 2020-08-03

**Authors:** Sam Passmore, Fiona M. Jordan

**Affiliations:** Department of Anthropology and Archaeology, University of Bristol, Bristol, UK

**Keywords:** Kinship, kinship terminology, cultural evolution, phylogenetics, language evolution

## Abstract

Kinship terminologies are the semantic systems of language that express kinship relations between individuals: in English, ‘aunt’ denotes a parent's sister. Theoretical models of kinship terminology diversity reduce over 10 billion possible organisations to six key types, each of which are hypothesised to be aligned with particular cultural norms of descent, marriage or residence patterns. Often, terminological type is used to infer social patterns in past societies based on these putative relationships between kinship terminologies and social structure, and these associations are staples of ‘Anthropology 101’. However, these relationships have not been scrutinised using modern comparative methods. Here we show that kinship terminologies vertically track language phylogeny in Austronesian, Bantu and Uto-Aztecan, three languages families of different time-depths and environments. We find no unidirectional or universal models of evolution in kinship terminology. Of 18 existing anthropological coevolutionary theories regarding kinship terminology and cultural practices across 176 societies, we find only patchy support, and no evidence for putative universal drivers of evolution in kinship terminologies.

## Graphical abstract

**Figure:**
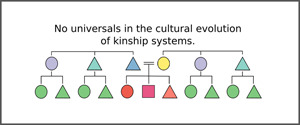

**Media Summary:** Kinship terminologies show no evidence for universal patterns of evolution, nor universal drivers of organisation.

## Introduction

**Figure 1.**
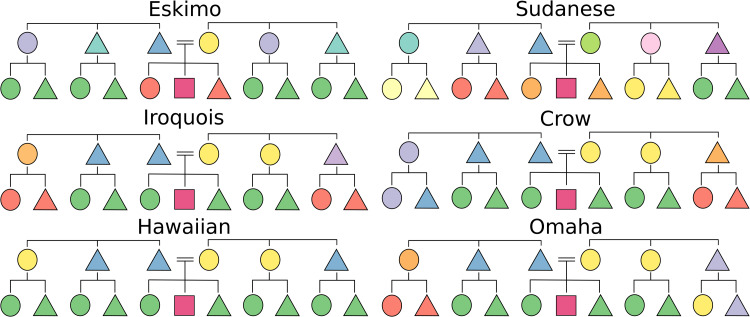
Six terminological types as formalised by Murdock, and named after societies in which they were first identified (Murdock, 1949). Triangles represent male, and circles female, relatives. The square represents Ego, the focal point of the terminology. Relatives are coloured to indicate where the same linguistic label (word) is used. Parallel lines indicate marriage.

All human societies recognise categories of kin with language that specifies how people are related. These categories are expressed linguistically in *kinship terminology*, a system of words for relatives. Globally, the patterning of these category systems is variable, yet it displays widespread typological convergence. Together with community norms of behaviour towards kin, kinship terminologies are fundamental aspects of human social diversity (Godelier, 2012). Over time, kinship organisation structures both cultural (Buckley & Boudot, 2017) and genetic (Lansing et al., 2017) diversity, so understanding the drivers of change in this domain provides insights into cultural evolution more generally. Despite the potential for unbridled variation, kinship terminologies are remarkably constrained. The universe of terminological systems for labelling siblings, cousins, parents and parent's siblings is 10,480,142,147 theoretically possible varieties (Nerlove & Romney, 1967). Yet historically, cross-cultural diversity in kinship terminology has been considered sufficiently represented by only a handful of major types, for example, the six-piece typology of cousin-organisation by Murdock (1949) (Figure 1).

Recent research shows that kinship terminologies may optimise between minimising cognitive load (simplicity) and reliably communicating intended meaning (informativeness) (Kemp & Regier, 2012). These constraints may account for limits on observed variation, but do not explain why we observe any variation at all, nor the origin and maintenance of particular variants. Experiments have demonstrated a cognitive bias for simplicity in kinship terminology and language learning more generally, which explains the absence of many theoretical possibilities, but does not explain why we observe differences in kinship terminology at all (S. Kirby, Tamariz, Cornish, & Smith, 2015; Rolando, Kirby, & Smith, 2018). This suggests that informativeness drives observed kinship terminological diversity, which is likely to be shaped by local cultural practices.

Anthropologists routinely propose that kinship categorisation is shaped by organisation of social structure, specifically, kinds of inheritance, descent, marriage and residence (Jones, 2010; Murdock, 1949). If norms of social structure act as global drivers of informativeness for kinship organisation, they therefore provide causal (although not necessarily deterministic) hypotheses of change about kinship terminologies (see Tables 1 and S3). Broadly speaking, social norms act to transmit cultural beliefs on (for example) sex, respect, obligation and the spatial proximity of kin, all of which affect and are affected by how kin are categorised. For example, nuclear family organisation might result in relatives outside this unit living at a distance, reducing the frequency of interactions and therefore the need to linguistically distinguish amongst types of relatives such as cousins, as in *Eskimo-types* and typical of English (Murdock, 1949) (Figure 1). Hypotheses of this sort stem from the accumulation of ethnographic observations or deductive reasoning, and are supported in the literature with simple cross-tabulations (Murdock, 1949). However, these hypotheses were derived using outmoded statistical tools and no longer represent the current state of kinship theory. It is increasingly clear that shared ancestry plays a significant role in explaining patterns of cultural diversity, not least in the domain of kinship (Guglielmino, Viganotti, Hewlett, & Cavalli-Sforza, 1995; Mace & Jordan, 2011). Much anthropological ‘common-knowledge’ concerning kinship is therefore subject to the problem of *phylogenetic autocorrelation*, where societies are related by common descent. Each society does not represent an independent occurrence of a phenomenon, thus violating assumptions of statistical independence (Naroll, 1965). Here we address these issues by using phylogenetic comparative methods (PCMs) to explore the cultural evolutionary patterns of kinship terminology diversity, particularly testing so-called drivers of change. This is not the first application of phylogenetic methods to kinship organisation; these approaches have been successfully applied within language families to understand ancestral states and feature-based patterns of change (Jordan, 2011). However, in this paper we present the first cross-language-family phylogenetic analysis of the drivers of kinship terminology.

Kinship terminology theory has developed as a specialty field away from the broad typological distinctions developed in the early eighteenth century, but Murdock's ideas are consistently revived in the broader anthropological sphere (Cronk & Gerkey, 2007; Guillon & Mace, 2016; Stone, 2014). While recent theorists have debated the biological underpinnings of kinship distinctions (Chapais, 2009); have explained terminology diversity by invoking constraint rules and optimality theory (Jones, 2010); and developed new approaches of kinship algebra (Read, 2013), the standard typology persists. Here we intend to systematically review the usefulness of this typology as a descriptive and predictive tool through systematic hypothesis testing using modern comparative methods. Our quantitative macro-evolutionary approach can contribute new insights to existing debates, arbitrate long-standing assertions with new methods and offer insight to underexplored areas of kinship diversity (Gray, Greenhill, & Ross, 2007).

To progress our understanding of kinship terminology evolution, we use language phylogenies in combination with ethnographic sources of cultural data to infer ancestral states, to estimate patterns of evolutionary change and to test for correlated evolution. Bayesian posterior samples of phylogenies mitigate and specify uncertainty about both the branching histories of populations as well as the evolutionary model of cultural change. We use data from D-PLACE (d-place.org) for kinship terminologies and social structure in three large language families (Austronesian, Bantu and Uto-Aztecan), totalling 176 societies (Gray, Drummond, & Greenhill, 2009; Grollemund et al., 2015; Kirby et al., 2016; Levinson, Greenhill, Gray, & Dunn, 2011; Murdock, 1967). These three families have different time depths and environmental histories, allowing us to probe universality in the patterns of change.

## Results and discussion

### Ancestral states

Tests for phylogenetic signal (vertical) and spatial (horizontal) autocorrelation demonstrate that the diversity of kinship terminologies in all language families is structured by shared ancestry (Table S4 in the Supporting Information). This justifies the use of PCMs. In Austronesian and Uto-Aztecan, shared ancestry is the clear driver of diversity. The separation of vertical and horizontal transmission is less clear in Bantu, suggesting that multiple processes may be acting in this language family.

Ancestral state inference of kinship terminology in each language family shows strong support for an *Eskimo-type* terminology at the root of the Austronesian language family (*P(Eskimo) = 0.847; 89% High density interval (HDI):* [*0.58, 1.00*]). In Bantu there is weak support for an *Iroquoian-type* root (*P(Iroquoian) = 0.395; HDI:* [*0.13, 0.67*]). We ‘fossilise’ the ancestral kinship state for Bantu and find weak evidence for an *Iroquoian-type* root over the next most likely state, *Hawaiian-type* (BF = 2.31) and all other possibilities, using the likelihood model comparison metric log Bayes Factors (BF) (Table S11). In Uto-Aztecan, we do not find outright support for any terminology, the most likely being *Hawaiian-type* (*P(Hawaiian) = 0.311; HDI:* [*0.23, 0.49*]). Fossilising ancestral states did not provide any hard evidence of an ancestral state. Details on model convergence diagnostics and baseline probabilities are provided in the Supporting Information.

In Austronesian languages, results can inform debate on kinship in Proto-Malayo-Polynesian (PMP), an early ancestral speech community c. 4 kya. The ‘bilateral hypothesis’ suggests that the ancestral state of PMP was *Hawaiian-type*, because many contemporary societies in the geographical south-east Asian home of PMP exhibit social structures that align with this kinship organisation, such as cousin incest taboos, limited polygyny and sexual equality (Murdock, 1949). The alternative ‘symmetric-exchange hypothesis’ suggests that ritualised marital exchange structured PMP society, rather than the features listed above (Lévi-Strauss, 1969; Wouden, 1968). Under the assumption of symmetric exchange, linguists reconstructed the PMP terminology to an *Iroquoian-type* (Blust et al., 1980). Using phylogenetic inference, we determine *Eskimo-type* as the most likely ancestral state (*P(Eskimo) = 0.897*).

Both the bilateral and systematic exchange hypotheses rely on certain assumptions. First, comparative ethnography accords preference to societies that are geographically close to family ‘homelands’ in inferring ancestral social structure. In contrast, PCMs use information from all societies in the family and let us explore a wider range of models of how social norms change. Second, kinship terminologies are assumed to be deterministically linked to social structure. Using PCMs, we show below that the relationship between terminologies and social structure is weak at best.

Previous phylogenetic reconstructions using the same data sources for kinship terminologies show that, in Bantu, *Hawaiian-type* terminologies are the most likely ancestral state, with some support for an *Iroquoian-type* (Guillon & Mace, 2016). In this study we use a newer phylogenetic posterior tree sample that contains more languages (see Supporting Information pg. 25). However, for both Guillon and Mace (2016) and our Bantu reconstructions, the likelihood of any particular terminology being ancestral is considerably lower than in Austronesian. This could be evidence of borrowing between Bantu societies, also supported by signal tests (Table S4), or it could reflect the relatively limited contemporary diversity in Bantu, i.e. the preponderance of *Iroquoian-type*. Borrowing may have occurred between Bantu societies, and/or with other non-Bantu sub-Saharan languages: we do not have methods to incorporate the latter in ancestral state inference. However, for example by analogy, genetic and linguistic evidence shows that Khoe-Sān women married into South-Western Bantu societies, following the patrilocal tradition commonly found in this region (Pakendorf, Gunnink, Sands, & Bostoen, 2017). Other papers have recorded systematic language borrowing throughout the Bantu family (Barbieri et al., 2014; Holden & Gray, 2006; Oliver, 1982). The uncertainty we see in Bantu ancestral state inference suggests that a multitude of cultural evolutionary processes (beyond stochastic change) are at work. Previous Bantu research suggests that shared ancestry is the pervading macro-evolutionary process through which kinship norms are transmitted (Guglielmino et al., 1995; Mulder, George-Cramer, Eshleman, & Ortolani, 2001); this makes sense if kinship is considered a ‘core’ cultural trait, and/or a semantic system sequestered from borrowing (Haspelmath & Tadmor, 2009). We know that many interdependencies are likely to drive cultural evolution (at minimum, shared ancestry and borrowing), but here we show their relative impact varies across language families.

Phylogenetic reconstruction is useful in regions where there has been less comparative research, such as Uto-Aztecan. Traditional linguistic reconstructions of Proto-Uto-Aztecan terms suggest a terminology system unlike any of the six common types, with a *Hawaiian-style* organisation in the generation of Ego, but unique terms for each member of the parental generation (G^+1^), consistent with a *Sudanese-type* (Shimkin, 1941). Our reconstruction finds *Hawaiian-type* cousin terms most likely, possibly reflecting the ego-generation-focused (G^0^) classification of kin terminologies used in our analyses, but we cannot interpret this with confidence. This type is technically agnostic with respect to G^+1^, but much writing on kinship assumes internal consistency between G^0^ to G^+1^ (Cronk, Steklis, Steklis, van den Akker, & Aktipis, 2018; Godelier, 2012). A drawback of phylogenetic modelling is that it only infers ancestral states that exist as observed states: the linguistic reconstruction of Proto-Uto-Aztecan as *Sudanese-styled* G^+1^ is compatible with our analyses because parental-generation variation is not captured in the data we use. This demonstrates two wider points: that internal variation exists within any existing kinship type (not all terminologies are ‘pure’), and that little systematic data on the global structure of that variation is available, including how societies transition between types (see below; Murdock, 1949). In this particular case the phylogenetic and linguistic reconstructions of Uto-Aztecan terminology suggest that kinship organisation may change in a more modular way than is currently being modelled with these data.

Our ancestral state analyses infer different basal terminologies (starting states) in each language family. They also show us that, while there is evidence of phylogenetic inheritance in the distribution of kinship terminologies, the lack of confidence in ancestral states suggests a second, non-inheritance process also acting on kinship organisation. We now briefly explore the potential for transitions between kinship terminology to follow some universal or generalising pattern, as seen in other semantic domains such as the evolution of colour terms (Berlin & Kay, 1991).

### Transition rates

**Figure 2.**
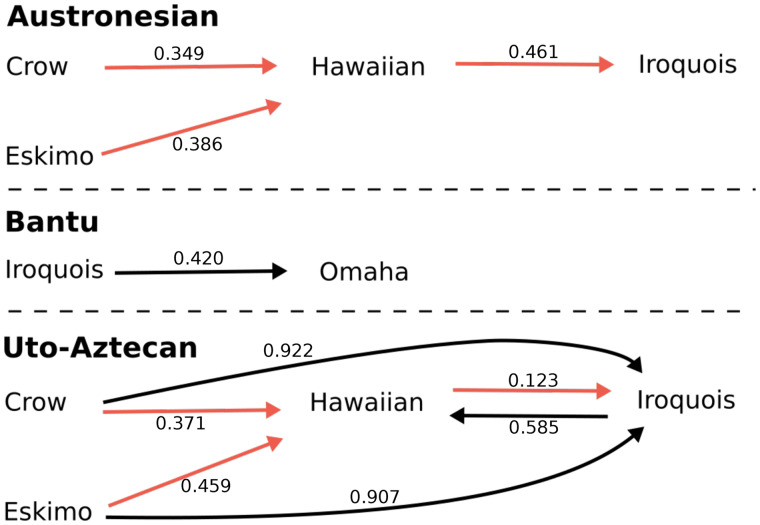
Transitions showing posterior–prior odds less than one for each language family. Red lines indicate repeated sub-graphs. Numbers attached to arrows are posterior-to-prior odds, the lower the value the stronger the evidence for a transition.Each language family shows distinct evolutionary patterns. The repeated sub-graph in Austronesian and Uto-Aztecan highlights some similarties, however transitions within Uto-Aztecan appear more flexible than in Austronesian, with three additional transitions - one of which allows for multi-directional change.

In ancestral state inference we estimate the direction and rate of change from one terminological type to another (a transition, Figure 2). We can use this information across language families to conclude whether kinship terminologies follow consistent patterns of change, or whether change is lineage-specific. Owing to the high number of potential transitions between six states, we use reversible-jump markov chain monte carlo (RJ-MCMC) methods, where negligible transitions are constrained to zero in order to avoid over parameterising the model. Statistically important transitions are determined via the posterior-to-prior odds following Currie et al. (2010). We calculate the prior likelihood of a single transition rate model and compare this with the number of times a particular transition is estimated in the posterior. Posterior-to-prior odds of less than 1 suggest that the parameter is unlikely to be equivalent to zero. Here we discuss all transitions with posterior-to-prior odds less than 1 in each language family. The odds of all transitions are shown in Tables S14–16. This is a very broad approach to analysing transitions, in that we only explore relatively extreme changes (from one organisation to another), and do not allow transitional types to exist. Previous work has explored patterns of evolutionary change using a feature-based approach, which offers a more detailed understanding of how kinship organisation changes over time (Jordan, 2011). Unfortunately, this data is not available in the cross-cultural setting used here, but the typology-level analysis should reveal high-level patterns of change, and whether we observe any similarities across language families.

In Austronesian, we see important shifts from *Crow-type* to *Hawaiian-type* (posterior-prior odds = 0.349), *Eskimo-type* to *Hawaiian type* (0.386), and *Hawaiian-type* to *Iroquois-type* (0.461). In Bantu, the only important shift we observe is from *Iroquois-type* to *Omaha-type* (0.420), with the next most likely transition being from *Iroquois-type* to *Hawaiian-type* (1.053), followed by *Omaha-type* to *Descriptive-type* (1.182). In Uto-Aztecan, we identify six important transitions: the first three are transitions to *Hawaiian-type* from *Iroquois-type* (0.123), *Crow-type* (0.371), and *Eskimo-type* (0.459). The second three are all transitions to *Iroquois-type* from *Hawaiian-type* (0.585), *Eskimo-type* (0.907) and *Crow-type* (0.922).

We observe one shared sub-graph between Austronesian and Uto-Aztecan: *Crow-type* and *Eskimo-type* transition to *Hawaiian-type*, and *Hawaiian-type* transitions to *Iroquois-type.* In Austronesian these are all of the important transitions, while in Uto-Aztecan we observe three additional transitions (see Figure 2). The shared sub-trajectory presents a potential bottom-up model for future research to pursue. Compared with existing models of change, we see support for transitions from *Crow-type* to *Hawaiian-type* (generational), but support for the opposite direction in the remaining transitions: *Eskimo-type* to *Hawaiian-type*, and *Hawaiian-type* to *Iroquois-type* (Kryukov, 1998).

A longstanding debate within terminology transitions is whether they are uni- or multi-directional (Trautman & Whitely, 2012). This is a complex question. Some theorists have argued strongly for a unidirectional pattern (Allen, 1989; Kryukov, 1998); however, more recent research is undecided (Trautman & Whitely, 2012). Evidence here shows that most change is unidirectional, but we do observe some evidence of multi-directional change in Uto-Aztecan, as well observing transitions that oppose existing models. Combining these pieces of evidence suggests that a multi-direction pattern of change is a more likely trajectory for kinship terminologies and is reflective of the adaptive relationship of kinship terminologies to local cultural, economic and ecological conditions.

If kinship terminologies were primarily linguistic outcomes of a cognitive communicative pressure for simplicity, our ancestral state and transition models could be expected to show more generalised patterns of change across all language families. Here, we only observe similarities between two languages families, and not identical patterns between these two. We might interpret the recurrence of the sub-graph as evidence of universal patterns of change, but we emphasise the importance of the additional transitions in Uto-Aztecan. While in Austronesian the sub-graph implies a unilineal trajectory, Uto-Aztecan models of change are much more flexible and highlight a unique and much more complicated model of evolution. A common perspective is that kinship terminologies are a ‘social-semantic product’ of complex and potentially locally specific adaptive pressures such as demography and resource control. The differences between languages families may be the result of these more local differences. We test this perspective by examining the most common broad-scale explanatory drivers of kinship terminology diversity, that terminologies are social-category responses to forms of social organisation

### Tests of co-evolution

We identify 18 theories within the anthropological literature which suggest that kinship terminologies are determined by patterns of social organisation: marriage, descent and residence (Murdock, 1949). When operationalised against available data, they total 29 separate statistical hypotheses of the correlates of kinship terminologies (Tables 1 and S3). Bayesian phylogenetic models of co-evolution allow us to test the evolutionary relationship between social structure and kinship terminologies, while controlling for patterns of shared ancestry. We run models of dependent and independent evolution and determine which model best fits the data using BFs. A BF greater than 10 indicates strong support for a dependent model of evolution, greater than 3 positive support and less than 3 no support (Kass & Raftery, 1995). To assess universality, hypotheses are tested in each family (Austronesian, Bantu, and Uto-Aztecan) as data allows, giving a total of 57 tests. We find some support for 14 of the 18 theories, but only 19 of the 29 statistical hypotheses. Only two theories are supported across two language families – and none in all three. Only 19 of 57 tests were supported overall, emphasising the lineage specific results of kinship terminological diversity.

#### Marriage

**Figure 3.**
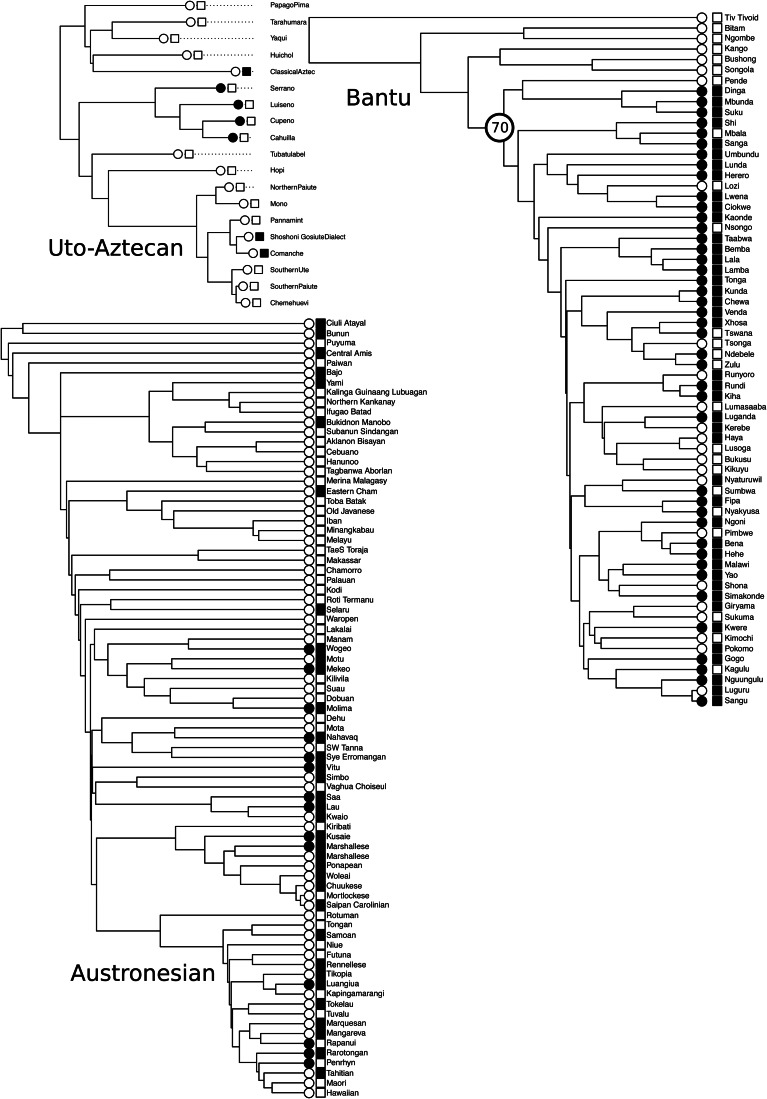
Indicative co-evolutionary relationship between Iroquois terminologies (circles) and preferential cross cousin marriage (squares). Black indicates the presence of a trait; white indicates absence. Dotted lines indicate non-contemporary societies. We find strong support for this relationship in Austronesian (BF = 9.79), and Bantu (BF = 13.85), but not in Uto-Aztecan. We also explored the dual inheritance of these traits in Bantu from node 70. All trees are maximum clade credibility trees.

These hypotheses include permitted or preferred cross-cousin marriage and the acceptance and rate of polygyny. If cross-cousin marriage is prescribed, a linguistic signal discerns marriageable and unmarriageable cousins, as in *Iroquoian-types* (Goody, 1970). We find positive evidence that *Iroquoian-types* co-evolve with the permitted and preferred cousin marriage (BF = 9.14 and BF = 9.79) in Austronesian, and strong evidence that they co-evolve with a preference for cousin marriage in Bantu (BF = 13.85). Despite the prevalence of this theory in historical literature, recent scholars do not believe *Iroquois-type* is compatible with cross-cousin marriage. Instead, it is the alternative *Dravidian-type* which is considered to fit with patterns of cross-cousin marriage (Trautmann & Barnes, 1998). This result suggests a need for investigation within Austronesian *Iroquoian-type* societies to determine whether they offer counterevidence for this theory or languages have been misclassified. Exploring the relationship between cross-cousin marriage and *Iroquois-types* in Bantu further, we see a particularly stable relationship between *Iroquoian-types* and preferential cross-cousin marriage that can be traced back to very early clades; this relationship is not present in the most early-branching societies in the group. Ancestral node 70 shows a high probability of an *Iroquoian-type* organisation and a preference for cross-cousin marriage (*P(Iroquoian & cross-cousin marriage) = 0.77*; see Figure 3), which has been inherited in 56% of descendant societies. Ancestral node 70 aligns with the Bantu expansion through the Savannah corridor ~4 kya, around modern Gabon and Republic of Congo (Grollemund et al., 2015). The Savannah corridor hypotheses proposes that climate change caused savannah habitats to encroach on the rainforest, guiding Bantu migration. It is plausible that the combination of environmental change and increased migration resulted in demographic change, influencing social structure and changing kinship organisation (Grollemund et al., 2015). Future research may be able to utilise models of maintenance in order to properly explore this proposal (Ross, Strimling, Ericksen, Lindenfors, & Mulder, 2016). In contrast, however, Uto-Aztecan societies in our sample with *Iroquoian-type* organisation never practice cross-cousin marriage, highlighting the lineage-specific processes apparent within our ancestral state analysis. It is also proposed that the prohibition of cousin marriage leads to the terminological conflation of all cousins, or cousins and siblings, as seen in *Eskimo-* and *Hawaiian-type*s (Goody, 1970). We found positive evidence of *Hawaiian-type* co-evolving with no preference for cousin marriage (BF = 5.42); however, in line with previous evidence, we find the no link between the prohibition of cross-cousin marriage and *Hawaiian-type* or *Eskimo-type* cousin organisation (Dole, 1972).

Polygyny acts to spatially separate lineal relatives, meaning women are surrounded by co-wives and not sisters, and children by half-siblings and not parallel cousins, which prevents the merging of lineal kinship terms (Murdock, 1949). This results in terminologies with different terms for parent's opposite sex siblings, as found in *Iroquois-* and *Crow*-*types*. We found weak support that Iroquoian types evolved with high rates of polygyny in Austronesian (BF = 2.53), but not low levels of polygyny. We find positive support for *Eskimo-types* co-evolving with monogamous marriage in Austronesian (BF = 7.36).

#### Residence

Recent phylogenetic models have shown residence patterns to evolve in lineage-specific ways (Moravec et al., 2018). Changes in residence patterns may shift kinship terminologies, because residence affects proximity and interaction with kin (Chapais, 2009). Avunculocal (living with a maternal uncle), patrilocal and matrilocal residence bring together lineal relatives in the parental generation (G^+1^) and are thought to result in terminologies which collapse parental terms with their same-sex siblings (e.g. father and father's brother). These are collectively described as unilocal residence, which are thought to correlate with *Iroquois*-, *Crow*- and *Omaha-types* (Murdock, 1949). Here, we analyse these collectively and independently. In Austronesian, we find strong support for avunculocal residence co-evolving with *Crow-types* (BF = 10.37), positive evidence for unilocal residence co-evolving with *Crow-types* (BF = 4.23), and positive evidence of unilocal residence co-evolving with *Iroquois-types* (BF = 6.44). In Bantu, we find positive evidence between *Iroquois-types* and matri-avunculocal residence (BF = 4.46), but no relationship between other patterns of unilocal residence and terminological types. Previous work has explored the relationship between *Iroquois-types* and unilocal residence in Bantu, and also found no relationship (Guillon & Mace, 2016). In Uto-Aztecan we find strong support for a relationship between *Iroquois-types* and unilocal residence (BF = 5.29). We find positive support for co-evolution between patrilocality and *Omaha-types* (BF = 6.42) in Bantu in contrast to previous analysis (Guillon & Mace, 2016), and between matrilocality and *Crow-types* (BF = 3.41), in Austronesian. However, when virilocality and uxorilocality are included under patri- and matrilocality, respectively, we only find positive evidence for matrilocality in Austronesian (BF = 2.5). Bilocal residence is thought to have the opposite effect to unilocality, bringing some collateral and some lineal relatives together through both sexes. This combination of relatives overrides the distinctions between groups and collapses terms into *Hawaiian-type* terminologies (Murdock, 1968). However, we find no support for *Hawaiian-types* and bilocal residence co-evolving, confirming previous phylogenetic results found in Bantu (Guillon & Mace, 2016). We extended this hypothesis to only include societies that reside with extended family groups but still found no support. *Eskimo-types* linguistically separate and emphasise the nuclear family, which is also perpetuated through neolocal residence. We find positive support for the relationship between Eskimo-types and neolocal residence (BF = 7.98), but not the cultural importance of nuclear families.

#### Descent

Social groups may align themselves based on common ancestry or descent, which increases the importance of specific kin-relations (also known as social differentials; Chagnon & Irons, 1979). Unilineal descent patterns prioritise one family line (paternal or maternal) over the other (Murdock, 1949). We find positive support for *Iroquois-types* and unilineal descent in Austronesian and Uto-Aztecan (BF = 7.10 and BF = 9.71). Where possible, we constrained this hypothesis to societies that also practised exogamy under a prediction from Murdock but found no support for this restricted test (Murdock, 1949). As with residence, the importance of distinguishing patri- and matrilineal relatives is supported: by a strong relationship in Austronesian with *Crow-types* and matrilineal descent (BF = 10.45), and positive evidence (BF = 3.34) of Bantu *Omaha-types* co-evolving with patrilineal descent (Goody, 1970), in contrast to Guillon and Mace's (2016) previous work. Bilineal societies do not promote the social differential of either lineage, and so are often associated with *Hawaiian-types*, which we find strong evidence for (BF = 10.70) in Austronesian (Murdock, 1970).

Overall, we find only some of these specific social norms to co-evolve with kinship terminology, largely specific to a single language family. Many relationships are not supported when accounting for shared ancestry. Some unsupported relationships may be ‘phylogenetically inert’ stable pairings of terminology and social structure, but we estimate this is only likely in three of 57 tests (see Table S84). We examined marriage, descent and residence as classic drivers; however, recent research has shown links between structural social change and religion (Watts, Sheehan, Atkinson, Bulbulia, & Gray, 2016), and land-tenure (Sheehan, Watts, Gray, & Atkinson, 2018). These moderating factors could influence kinship organisational change and be tested in the future.

We are confident these results accurately model the available data, but we do not conclude that earlier anthropological work was mistaken in postulating social norms as drivers of terminological change. Instead, we suggest that these results reflect the explanatory limits of the existing typologies. We hope that this paper highlights the inadequacies of the Murdock typology as a tool used to infer behavioural organisations, but also want to highlight its inability to accurately describe global variation. For example, Lozi (Western Zambia) and Tongan (Polynesia) have kinship terminologies which are both classified as *Hawaiian-type*. Lozi distinguish cousins based on their sex, while Tongans distinguish cousins based on the sex relative to the speaker. Using more granular data we could explore the structure of the typological variation, identify subtle but important differences in kinship types across language families or identify paths of evolution that result in convergent terminological types.

**Table 1.** All tests of co-evolution between terminologies and social structure

Hypothesis	AN	BT	UA
Crow and polygyny (high)	1.059		
Crow and matri-avunculocal residence	**10**.**374**	−1.536	
Crow and matrilineal	**10**.**45**	−1.82	
Crow and matrilocality	**3**.**401**	0.753	
Crow and polygyny	−1.839		
Crow and unilocal residence	**4**.**228**	−2.231	
Eskimo and absence of permitted cousin-marriage	−0.323		
Eskimo and absence of preferential cousin-marriage	−2.423		
Eskimo and bilineal descent	−1.634		
Eskimo and monogamy	**7**.**368**		
Eskimo and neolocal residence	**7**.**984**		
Eskimo and nuclear families	0.963		
Hawaiian and absence of permitted cousin-marriage	0.211	−2.07	−1.114
Hawaiian and absence of preferential cousin-marriage	**5**.**421**	−2.77	−1.632
Hawaiian and bilineal descent	**10**.**7**	−1.44	−1.567
Hawaiian and bilocal extended family	0.945	−1.756	−0.298
Hawaiian and bilocal residence	−1.547	−2.097	−0.171
Iroquois and permitted cross-cousin marriage	**9**.**135**	0.132	−1.607
Iroquois and preferential cross-cousin marriage	**9**.**785**	**13**.**49**	−1.189
Iroquois and unilineal descent with exogamy	0.205	−2.012	−1.319
Iroquois and polygyny (high)	**2**.**528**		1.854
Iroquois and matri-avunculocal residence	−3.243	**4**.**458**	−1.269
Iroquois and polygyny	−1.425		
Iroquois and unilineal descent	**7**.**101**	−1.586	**9**.**705**
Iroquois and unilocal residence	**6**.**441**	−2.486	**5**.**29**
Omaha and matri-avunculocal residence		0.232	
Omaha and patrilineal descent		**3**.**336**	
Omaha and patrilocal residence		**6**.**424**	
Omaha and unilocal residence		−2.09	

Each row indicates a statistical hypothesis, and columns indicate the language family in which the test is performed, with cells containing log Bayes factors: BF < 2 indicates weak evidence, >2 positive evidence, 5–10 strong evidence and >10 very strong evidence. Figures in bold show results with log Bayes factors two and above. Where there was no data available to test the hypotheses, the cell is left blank. See Table S3 for the source and quote for each hypothesis. AN, Austronesian; BT, Bantu; UA, Uto-Aztecan.

## Conclusion

We have analysed kinship typology across three language families of different time-depths and environments and have been unable to discover strong universal drivers or unidirectional patterns of evolution in kinship terminology types. Partially, we attribute this to the insufficiency of the typology in representing global kinship terminological diversity at the right level of detail. For ancestral state reconstruction and transitions, the typology offers a restrictive view of change. It fails to incorporate within-type variation, or between-type variation by only allowing transitions between attested fixed states. Our co-evolutionary results may similarly be considered as the result of insufficient specification by the Murdock typology. However, we note that in the literature underlying the set of hypotheses tested here, scholars proposed or observed these associations *using this typology*: it may be a clumsy categorical tool, but it was the framework within which these anthropological relationships were largely proposed. More constructively, it is possible that many of the social-sematic relationships that we tested were not supported because the kinship terminologies contain sub-types, ‘hidden’ through categorisation in Murdock's scheme. For example, the disentanglement of *Iroquoian-type* and *Dravidian-type* identified key linguistic differences that made opposite predictions concerning cross-cousin marriage (discussed above; Trautmann & Barnes, 1998). The academic utility of this typology may have reached its limits. Future research to explore the existence of sub-types or variants will require comprehensive lexical data. We are at present building such a ‘KinBank’ database across multiple language families. Fine-grained complete terminological data can be coded to represent key social and linguistic distinctions, allowing us to more realistically characterise types and patterns of change by focusing on the reconstruction and transitions of features (such as age or gender; see Jordan, 2011).

Our key message here is that universal relationships linking kinship terminology and social structure are not supported. This is not because one or two societies do not follow a trend: for most of the hypotheses tested, we could not conservatively even claim ‘statistical regularity’. Cross-cultural universals have come under increased scrutiny as improved data are more readily available (Kirby et al., 2016) and phylogenetic methods are adapted for cultural questions. For example, similar methodological studies show lineage-specific processes of word-order evolution across Indo-European, Austronesian, Bantu and Uto-Aztecan (Dunn, Greenhill, Levinson, & Gray, 2011), and lineage-specific transitions in post-marital residence in the above language families plus Pama–Nyungan (Moravec et al., 2018). Testing of these longstanding assertions about cultural change has been enabled by accessible ethnographic data and language phylogenies (Kirby et al., 2016), and these results clear the ground for more detailed characterisations of kinship organisation in future.

## Materials and methods

### Data and phylogenies

We selected Austronesian, Bantu and Uto-Aztecan language families because of their large size and cultural data availability. These languages families cover 14% of the societies in D-PLACE, and 20% of phylogenetic diversity, giving different evolutionary time depths (Austronesian = 5.5 ka; Bantu = 4 ka; Uto-Aztecan = 5 ka), geographic environments and ecological pressures. Cultural data was paired with the most recent published phylogenies for Austronesian (Gray et al., 2009), Bantu (Grollemund et al., 2015) and Uto-Aztecan (Levinson et al., 2011) linguistic phylogenies. Posterior samples of 1000 phylogenies were used in all cases. All terminological and social data was taken from the Ethnographic atlas subset of D-PLACE (dplace.org) (K. R. Kirby et al., 2016; Murdock, 1967). Any societies with missing data were pruned. Co-evolutionary methods required binary data: see Table S2 for coding decisions for data availability in each hypothesis. Longitude and latitude for each society were also taken from D-PLACE. Hypotheses were found through exploration of the literature and using the ‘Explaining Human Culture’ database (Ember, 2018).

### Signal tests

We performed five tests to assess whether shared ancestry was a constraint on kinship diversity. The phylogenetic ‘D’ test uses simulation to determine whether the clustering of binary variables on a phylogeny follow patterns of Brownian motion (*D* = 0 indicates perfectly Brownian clusters and *D* < 0 strong clustering) or random clustering (*D* = 1 indicates complete randomness). Mantel tests were used to assess geographic signal. Mantel tests use random permutation and Pearson's correlation statistics to determine the correlation between two matrices. We compared log geographic distance, calculated with the Haversine formula, with a binary similarity matrix of each terminology present in each language family, for 999 permutations. Phylogenetic distance was calculated using cophenetic distance, and the cophenetic function in base R (R Core Team, 2018). To determine whether phylogenetic or geographic distances best determined the distribution of terminologies, we used partial Mantel tests on these matrices (terminology, geographic and phylogenetic distance).

### Ancestral state inference

We estimate the probability of a particular terminology at the root of each language phylogeny and the patterns of change between each state. We perform Bayesian reversible-jump MCMC ancestral reconstructions of kinship terminologies for each language family using BayesTraits V3.0 Multistate (Pagel & Meade, 2017). Multistate uses a posterior of phylogenies to estimate the probability of each terminology present in the taxa at the phylogeny root, and an estimation of the rate (*Q*) matrix. The reversible-jump approach searches the model space for an optimal solution by dynamically setting some rate parameters to zero. The *Q* matrix shows the likelihood of changing from any state to any other. MCMC chains were run for 10^9^ iterations, sampling every 50,000 iterations with a burn-in of 10,000 iterations, resulting in a posterior of 19,999 iterations. A stepping stone sampler was used to estimate the marginal likelihood. For all trees we use an exponential prior (*λ* = 10). We used 100 stones sampled every 1000 iterations. Each analysis ran three times to test consistent MCMC convergence using the Gelman–Rubin diagnostic (Gelman & Rubin, 1992). Owing to uncertainty in the reconstruction of the Bantu ancestral state, each possible taxon was fossilised as the root to estimate likelihoods. We calculated pairwise BF to assess the evidence for each response. Fossilised ancestral state reconstruction was used on the Bantu language family in an attempt to parse a more likely ancestral state. We forced the model to assume an ancestral state and used model comparison to determine the evidence for each possible ancestral state.

### Co-evolutionary tests

Co-evolutionary tests were performed by comparing dependent and independent phylogenetic Bayesian RJ-MCMC models of evolution using BayesTraits V3.0 Discrete. See Supporting Information section 4.1 for number of iterations, burn-in length, sampling rate and priors. Each analysis ran three times to ensure consistent MCMC convergence and used Gelman–Rubin diagnostics. The posterior from the first sample is presented as our results. We preferred the dependent or independent model of co-evolution based on log BFs.

## Research transparency and reproducibility

The data, scripts, output files and Supporting Information summarising these files can be found at https://doi.org/10.5281/zenodo.3269428.

## Supplementary material

For supplementary material accompanying this paper visit http://dx.doi.org/10.1017/ehs.2020.41. click here to view supplementary material
