# Supplementary material for: No universals in the cultural evolution of kinship terminology
Source: Evol Hum Sci. 2020 Aug 3;2:e42. doi: 10.1017/ehs.2020.41 (PMC7612818; doi:10.1017/ehs.2020.41)
Supplement: Supplementary file 1 [file S2513843X20000419sup001.docx]

Supplementary Material

Sam Passmore and Fiona Jordan

25 June 2020

Table of Contents

[Data 1](#_Toc29196004)

[Kinship and social structure 1](#_Toc29196005)

[Co-evolutionary hypotheses and references 3](#_Toc29196006)

[Phylogenies 12](#_Toc29196007)

[Signal tests 12](#_Toc29196008)

[Ancestral state 16](#_Toc29196009)

[MCMC review 16](#_Toc29196010)

[Fossilized Bantu 17](#_Toc29196011)

[Fossilized Uto-Aztecan 18](#_Toc29196012)

[PMP in Austronesia 19](#_Toc29196013)

[Transition rates 19](#_Toc29196014)

[Guillon and Mace comparison 22](#_Toc29196015)

[Co-evolution tests 23](#_Toc29196016)

[MCMC settings 23](#_Toc29196017)

[MCMC review and Bayes Factors 27](#_Toc29196018)

[Bantu node 70 state inference 73](#_Toc29196019)

[Multiple comparisons 74](#_Toc29196020)

[Phylogenetic Inertia 74](#_Toc29196021)

[References 77](#_Toc29196022)

# Data

## Kinship and social structure

All data was extracted from the D-PLACE Ethnographic atlas [GitHub repository](https://github.com/D-PLACE/dplace-data). Counts of the number of societies used for the signal and ancestral state analyses for each language family are in table S1. Question and variable codes are displayed below in table S2, frequency of terminology types are show in figure 2. Due to the nature of the analysis, all variables are binary coded. The numbers in the value columns indicate the criteria for a society to have the terminology or social structure coded as present. For details on what each code indicates, refer to the Ethnographic Atlas codebook or the [D-PLACE GitHub repository](https://github.com/D-PLACE/dplace-data/blob/master/datasets/EA/codes.csv).

Table S1: Count of societies in signal test and ancestral state analyses, by language family

|  | Count |
| --- | --- |
| Austronesian | 85 |
| Bantu | 69 |
| Uto-Aztecan | 22 |
| 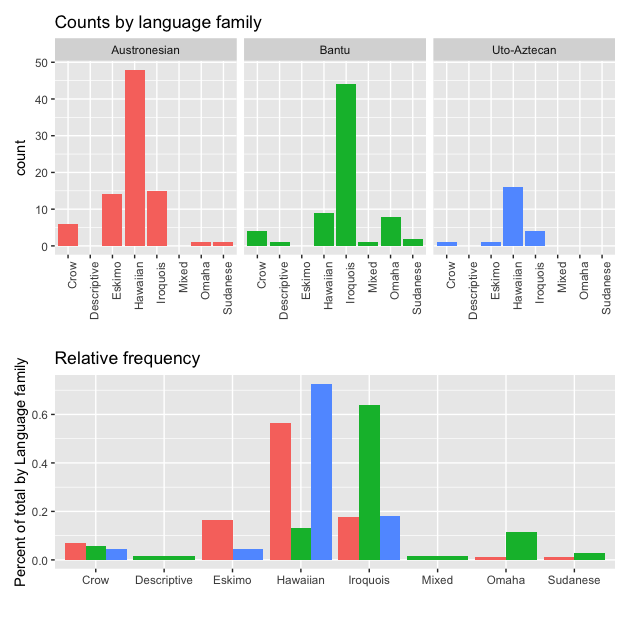 |  |
| *Figure S1: The count and relative frequency of terminologies in each language family.* |  |

Table S2: Data coding taken from D-PLACE and Co-evolutionary analyses data counts

| terminology | question | value | social structure | question | value | AN | BT | UA |
| --- | --- | --- | --- | --- | --- | --- | --- | --- |
| Crow | 27 | 1 | matrilineal | 43 | 3 | 85 | 69 |  |
| Crow | 27 | 1 | high.polygyny | 9 | 3,4,5,6 | 80 |  |  |
| Crow | 27 | 1 | polygyny | 9 | 2,3,4,5,6 | 80 |  |  |
| Crow | 27 | 1 | matri.anvunclocalresidence | 10 | 1,5,9 | 84 | 69 |  |
| Crow | 27 | 1 | uni.localresidence | 10 | 1,5,8,9,10 | 84 | 69 |  |
| Eskimo | 27 | 3 | bi.linealdescent | 43 | 2,5,7 | 85 |  |  |
| Eskimo | 27 | 3 | uni.linealdescent | 43 | 1,3,4 | 85 |  |  |
| Eskimo | 27 | 3 | absenceofcousinmarriage.permitted | 25 | 10,11,15 | 79 |  |  |
| Eskimo | 27 | 3 | absenceofcousinmarriage.preference | 23 | 7,8,11,12 | 79 |  |  |
| Eskimo | 27 | 3 | monogamy | 9 | 1 | 80 |  |  |
| Eskimo | 27 | 3 | neo.localresidence | 10 | 6 | 84 |  |  |
| Eskimo | 27 | 3 | nuclear.families | 8 | 1,2 | 83 |  |  |
| Hawaiian | 27 | 4 | bi.linealdescent | 43 | 2,5,7 | 85 | 69 | 23 |
| Hawaiian | 27 | 4 | bi.localextendedfamily | 8 | 6,7,8 | 83 | 69 | 22 |
| Hawaiian | 27 | 4 | absenceofcousinmarriage.permitted | 25 | 10,11,15 | 79 | 66 | 19 |
| Hawaiian | 27 | 4 | absenceofcousinmarriage.preference | 23 | 7,8,11,12 | 79 | 66 | 19 |
| Hawaiian | 27 | 4 | bi.localresidence | 10 | 2,11,12 | 84 | 69 | 22 |
| Iroquois | 27 | 5 | exogamy.unilineal.descent | 15 | 4 | 78 | 62 | 19 |
| Iroquois | 27 | 5 | uni.linealdescent | 43 | 1,3,4 | 85 | 69 | 23 |
| Iroquois | 27 | 5 | cross.cousinmarriage.permitted | 25 | 1,6,9 | 79 | 66 | 19 |
| Iroquois | 27 | 5 | cross.cousinmarriage.preferred | 23 | 1,2,3,5,6,8,12,13,14 | 79 | 66 | 19 |
| Iroquois | 27 | 5 | high.polygyny | 9 | 3,4,5,6 | 80 |  | 22 |
| Iroquois | 27 | 5 | polygyny | 9 | 2,3,4,5,6 | 80 |  |  |
| Iroquois | 27 | 5 | matri.anvunclocalresidence | 10 | 1,5,9 | 84 | 69 | 22 |
| Iroquois | 27 | 5 | uni.localresidence | 10 | 1,5,8,9,10 | 84 | 69 | 22 |
| Omaha | 27 | 6 | patrilineal | 43 | 1 |  | 69 |  |
| Omaha | 27 | 6 | matri.anvunclocalresidence | 10 | 1,5,9 |  | 69 |  |
| Omaha | 27 | 6 | uni.localresidence | 10 | 1,5,8,9,10 |  | 69 |  |
|  |  |  |  |  |  |  |  |  |
|  |  |  |  |  |  |  |  |  |
|  |  |  |  |  |  |  |  |  |

###

### Co-evolutionary hypotheses and references

Table S3: hypotheses from earliest found source with quotes, references and page numbers

| terminology | hypotheses | quote | justification | reference |
| --- | --- | --- | --- | --- |
| Crow | High polygyny | “Non-sororal polygyny tends to be associated with kinship terminology of the bifurcate collateral type” | Polygyny spatially separates lineal relatives, meaning women are surrounded by co-wives and not sisters, and children by half-siblings and not parallel cousins, which prevents merging of lineal kinship terms. | Murdock, G. P. (1949). Social structure (Vol. xvii). Oxford, England: Macmillan. |
| Crow | Matri-avunculocal residence | “Crow systems should occur more frequency in societies which are avunculocal …” | Avunculocal residence means married couples move to live with the husband’s mother’s brother and are spatially closer to maternal kin, meaning there is more pressure to specifically identify maternal kin, and paternal kin can be grouped together. With specific reference to Crow societies, it is likely that this also aligns with matrilateral cross-cousin marriage. | Eyde, D. B., & Postal, P. M. (1961). Avunculocal and Incest: The Development of Unilateral Cross-Cousin Marriage and Crow-Omaha Kinship Systems. American Anthropologist, 63(4), 747–771. |
| Crow | Matrilineal | “The tables show a strong correlation between Omaha terms and patrilineal unilineal descent groups and between Crow terms and matrilineal unilineal descent groups” | A matrilineal descent system imposes a higher social status (social differential) on maternal kin, and therefore the need to distinguish maternal kin over paternal. This allows the grouping of paternal kin. | Goody, J. (1970). Cousin Terms. Southwestern Journal of Anthropology, 26(2), 125–142. |
| Crow | Polygyny | “Non-sororal polygyny tends to be associated with kinship terminology of the bifurcate collateral type” | Polygyny spatially separates lineal relatives, meaning women are surrounded by co-wives and not sisters, and children by half-siblings and not parallel cousins, which prevents merging of lineal kinship terms. | Murdock, G. P. (1949). Social structure (Vol. xvii). Oxford, England: Macmillan. |
| Crow | Uni-local residence | “In the presence of patrilocal, matri-local or avunculocal residence, separate terms tend to be applied to relatives of the same generation who are linked to ego through connecting relatives of a different sex.” | A unilocal residence rule brings into proximity a group of relatives linked under one line of decent. This rule acts as a social differential to support bifurcation of cousin terms. | Murdock, G. P. (1949). Social structure (Vol. xvii). Oxford, England: Macmillan. |
| Eskimo | Absence of CM permitted | “Prohibition on cousins vary [in Eskimo-types], but as one might expect, are inevitably bilateral” | If all cousins are unmarriageable, there is no social differential and therefore no need to distinguish them | Fox, R. (1967). Kinship and Marriage: An Anthropological Perspective. Cambridge University Press. |
| Eskimo | Absence of CM preference | “Prohibition on cousins vary [in Eskimo-types], but as one might expect, are inevitably bilateral” | If all cousins are unmarriageable, there is no social differential and therefore no need to distinguish them | Fox, R. (1967). Kinship and Marriage: An Anthropological Perspective. Cambridge University Press. |
| Eskimo | Bi-lineal descent | “…there is a distinct association of Eskimo terms with bilateral [descent] systems” | When terms differentiate between full siblings and cousins when they are socially differentiated (in this case it is proposed to reflect direct patterns of inheritance). | Goody, J. (1970). Cousin Terms. Southwestern Journal of Anthropology, 26(2), 125–142. |
| Eskimo | Monogamy | “The following variables are dependent upon diverging devolution (transmission of property to both males and females): greater control of women’s’ marriage (prohibition of premarital sex, endogamy, father’s brother’s daughter marriage, monogamy, alternative residence) and by extension Eskimo kinship terminology which isolates the sibling group form “cousins"" | When there is a social focus on the monogamous nuclear family, there is a need to distinguish children and siblings from other closely related nuclear families. | Goody, J., Irving, B., & Tahany, N. (1970). Causal Inferences Concerning Inheritance and Property. Human Relations, 24, 295–314. |
| Eskimo | Neo-local residence | “Neolocal residence tends to be associated with kinship terminology of the lineal type.” | Neo-local residence results in the spatial separation of lineal relatives from all collateral relative, and emphasizes the residential group | Murdock, G. P. (1949). Social structure (Vol. xvii). Oxford, England: Macmillan. |
| Eskimo | Nuclear families | “In the absence of clans and polygamous and extended families, the isolated nuclear family tends to be associated with kinship terminology of the lineal type.” | The isolation of the nuclear family operates as a social differential and favours separate terms for the lineal and collateral kin. | Murdock, G. P. (1949). Social structure (Vol. xvii). Oxford, England: Macmillan. |
| Hawaiian | Absence of CM permitted | “Hawaiian [kin] terms … are associated with the prohibition on [cross] cousin marriage” | If all cousins are unmarriageable, there is no social differential and therefore no need to distinguish them | Goody, J. (1970). Cousin Terms. Southwestern Journal of Anthropology, 26(2), 125–142. |
| Hawaiian | Absence of CM preference | “Hawaiian [kin] terms … are associated with the prohibition on [cross] cousin marriage” | If all cousins are unmarriageable, there is no social differential and therefore no need to distinguish them | Goody, J. (1970). Cousin Terms. Southwestern Journal of Anthropology, 26(2), 125–142. |
| Hawaiian | Bi-lineal descent | “Bilateral kindreds tend to be associated with kinship terminology of the generation type” | If there is no social differential between maternal or paternal relatives, nor is there a pressure to distinguish nuclear family from other kin, then there is no pressure to linguistically distinguish siblings and cousins, or parents, and parent’s siblings. | Murdock, G. P. (1949). Social structure (Vol. xvii). Oxford, England: Macmillan. |
| Hawaiian | Bi-local extended family | “The several types of extended family … depend upon the prevailing rule of residence and exert the influences upon kinship nomenclature already set forth in Theorems 6 and 11” | Extended families increase social participation and interdependence within society and therefore emphasise the effect of bi-lineal descent. | Murdock, G. P. (1949). Social structure (Vol. xvii). Oxford, England: Macmillan. |
| Hawaiian | Bi-local residence | “Bi-local residence tends to be associated with kinship terminology of the generation type” | Bi-lineal decent brings together some lineal kin, and some collateral kin, some through men and some though women. All these groups combined are then all overridden, and individuals are treated equally. | Murdock, G. P. (1949). Social structure (Vol. xvii). Oxford, England: Macmillan. |
| Iroquois | Cross CM permitted | “Iroquois [kin] terms … are associated with preferred cross-cousin marriage” | If cousin marriage is allowed (or preferred) in some but not all cousins, there is a pressure to linguistically distinguish those who are marriageable from those who are not. | Goody, J. (1970). Cousin Terms. Southwestern Journal of Anthropology, 26(2), 125–142. |
| Iroquois | Cross CM preferred | “Iroquois [kin] terms … are associated with preferred cross-cousin marriage” | If cousin marriage is allowed (or preferred) in some but not all cousins, there is a pressure to linguistically distinguish those who are marriageable from those who are not. | Goody, J. (1970). Cousin Terms. Southwestern Journal of Anthropology, 26(2), 125–142. |
| Iroquois | Exogamy & unilineal descent | “In the presence of exogamous matrilineal or patrilineal lineages, sibs, phratries, or moieties, terms for lineal relatives tend to be extended, within the same sex and generation, to collateral kinsmen who would be affiliated with them under either unilinear rules of descent.” | Kin that are members of the same exogamous unilinear group are socially equalized with collateral kin and are therefore terms are merged. | Murdock, G. P. (1949). Social structure (Vol. xvii). Oxford, England: Macmillan. |
| Iroquois | High polygyny | “Non-sororal polygyny tends to be associated with kinship terminology of the bifurcate collateral type” | Polygyny spatially separates lineal relatives, meaning women are surrounded by co-wives and not sisters, and children by half-siblings and not parallel cousins, which prevents merging of lineal kinship terms. | Murdock, G. P. (1949). Social structure (Vol. xvii). Oxford, England: Macmillan. |
| Iroquois | Matri-avunculocal residence | “Matrilocal and avunculocal residence tend to be associated with kinship terminology of the bifurcate merging type” | Avunculocal residences aligns relatives so that the distinction of collaterally is minimized and there is a tendency to extend kinship terms from lineal to collateral co-residing relatives. | Murdock, G. P. (1949). Social structure (Vol. xvii). Oxford, England: Macmillan. |
| Iroquois | Polygyny | “Non-sororal polygyny tends to be associated with kinship terminology of the bifurcate collateral type” | Polygyny spatially separates lineal relatives, meaning women are surrounded by co-wives and not sisters, and children by half-siblings and not parallel cousins, which prevents merging of lineal kinship terms. This results in different terms for parent’s opposite sex sibling terms. | Murdock, G. P. (1949). Social structure (Vol. xvii). Oxford, England: Macmillan. |
| Iroquois | Uni-lineal descent | “The classic but erroneous anthropological view concerning the nature of the ‘Iroquois type’ of kinship ­ … is that this kind of system classifies kin by membership in unilineal descent groups” | ? | Lounsbury, F. G. (1964). The structural analysis of kinship semantics. Mouton. |
| Iroquois | Uni-local residence | “In the presence of patrilocal, matri-local or avunculocal residence, separate terms tend to be applied to relatives of the same generation who are linked to ego through connecting relatives of a different sex.” | A uni-local residence rule brings into proximity a group of relatives linked under one line of decent. This rule acts as a social differential to support bifurcation of cousin terms. | Murdock, G. P. (1949). Social structure (Vol. xvii). Oxford, England: Macmillan. |
| Omaha | High polygyny | “Non-sororal polygyny tends to be associated with kinship terminology of the bifurcate collateral type” | Polygyny spatially separates lineal relatives, meaning women are surrounded by co-wives and not sisters, and children by half-siblings and not parallel cousins, which prevents merging of lineal kinship terms. This results in different terms for parent’s opposite sex sibling terms. | Murdock, G. P. (1949). Social structure (Vol. xvii). Oxford, England: Macmillan. |
| Omaha | Matri-avunculocal residence | “Matrilocal and avunculocal residence tend to be associated with kinship terminology of the bifurcate merging type” | Avunculocal residences aligns relatives so that the distinction of collaterally is minimized and there is a tendency to extend kinship terms from lineal to collateral co-residing relatives. | Murdock, G. P. (1949). Social structure (Vol. xvii). Oxford, England: Macmillan. |
| Omaha | Patrilineal | “The tables show a strong correlation between Omaha terms and patrilineal unilineal descent groups and between Crow terms and matrilineal unilineal descent groups” | Patrilineal descent acts as a social differential between maternal and paternal kin, allowing more terminological distinction between patrilineal relatives, and grouping of matrilineal relatives. | Goody, J. (1970). Cousin Terms. Southwestern Journal of Anthropology, 26(2), 125–142. |
| Omaha | Polygyny | “Non-sororal polygyny tends to be associated with kinship terminology of the bifurcate collateral type” | Polygyny spatially separates lineal relatives, meaning women are surrounded by co-wives and not sisters, and children by half-siblings and not parallel cousins, which prevents merging of lineal kinship terms. This results in different terms for parent’s opposite sex sibling terms. | Murdock, G. P. (1949). Social structure (Vol. xvii). Oxford, England: Macmillan. |
| Omaha | Uni-local residence | “In the presence of patrilocal, matri-local or avunculocal residence, separate terms tend to be applied to relatives of the same generation who are linked to ego through connecting relatives of a different sex.” | A uni-local residence rule brings into proximity a group of relatives linked under one line of decent. This rule acts as a social differential to support bifurcation of cousin terms. | Murdock, G. P. (1949). Social structure (Vol. xvii). Oxford, England: Macmillan. |

##

## Phylogenies

We tested hypotheses across three languages families, where data allowed. This was the Austronesian, Bantu, and Uto-Aztecan language phylogenies (Dunn et al., 2011; Gray et al., 2009; Grollemund et al., 2015). All language to phylogeny pairings were taken from decisions made in Kirby et al. (2016). In Austronesian, we sub-sampled 1000 phylogenies from a posterior sample of 4199 phylogenies. Austronesian phylogenies were estimated through linguistic data and supported by genetic evidence, and archaeological records. Detailed methods can be found in Gray et al. (2009). Trees were pruned from the original sample of 400 languages, to 80 languages based on data availability. In Bantu, we sampled 1000 from a posterior sample of 2000 phylogenies developed in Grollemund et al. (2015). These trees were built using linguistic data and calibrated using the archaeological record. Trees were pruned from the original sample of 425 languages, to 69 languages based on data availability. In Uto-Aztecan, we sampled 1000 phylogenies from a posterior of 10,000 phylogenies modeled by Dunn et al. (2011). This posterior was built using linguistic data. Trees were pruned from the original sample of 34 languages, to 19 based on data availability. All branch lengths are standardized to have a mean length of 0.1, as per BayesTraits recommendations.

# Signal tests

We performed 4 signal tests to assess the hypotheses that shared-ancestry was a constraint on kinship diversity. The phylogenetic ‘D’ test uses simulation to determine whether the clustering of binary variables on a phylogeny follow patterns of Brownian motion (D = 0 indicates perfectly Brownian clusters and D < 0 strong clustering) or random clustering (D = 1 indicates complete randomness). To test whether geography may also predict the distribution of terminologies, we used Mantel tests. Mantel tests use random permutation and Pearson’s correlation statistics to determine the correlation between two matrices. Here we compare log geographic distance, calculated with the Haversine formula, to a binary similarity matrix of each terminology present in each language family, each over the default setting of 999 permutations. To determine whether phylogenetic or geographic distances best determined the distribution of terminologies, we used partial Mantel tests. Partial Mantel tests control for a confounding third matrix, while comparing another two matrices. We perform a test between a terminology and geographic distance, controlling for phylogenetic distance, and another between terminology and phylogenetic distance, controlling for geographic distance. Phylogenetic distance is calculated using cophenetic distance, and the cophenetic function in R {stats} (R Core Team 2018). Table S4 shows the mean posterior result from 1000 phylogenies for all terminologies present within each language family. However, we only consider results viable if the terminology consists of more than 10% of the overall sample. All p-values are Bonferroni corrected.

The code for all signal tests can be found in file analysis/signal-tests.R. This file comes with helper functions analysis/signal-functions and analysis/mantel-functions for the D-statistic and Mantel tests respectively.

Table S4: Signal tests for each terminology within each language family. Columns 2 and 3 indicate the presence and absence of a terminology. Columns 4 to 6 are outputs from the D-statistic test. Columns 7 to 21 are output from Mantel tests. P-values and Bonferroni corrected values shown

|  | Terminology | Absent | Present | D | P. Brownian | P. Random | Z-stat | p-value | bonf-p | Z-stat | p-value | bonf-p | Z-stat | p-value | bonf-p | Statistic | p-value | bonf-p | Statistic | p-value | bonf-p |
| --- | --- | --- | --- | --- | --- | --- | --- | --- | --- | --- | --- | --- | --- | --- | --- | --- | --- | --- | --- | --- | --- |
| Austronesian | Crow | 79 | 6 | -0.115 | 0.603 | 0.036 | 1752616.53 | 0.039 | 0.233 | 3789.807 | 0.400 | 1.000 | 32.773 | 0.028 | 0.167 | 0.012 | 0.368 | 1.000 | -0.116 | 0.977 | 1.000 |
|  | Eskimo | 71 | 14 | -0.494 | 0.806 | 0.000 | 4905208.29 | 0.219 | 1.000 | 8155.126 | 0.180 | 0.985 | 78.033 | 0.038 | 0.229 | -0.004 | 0.500 | 1.000 | 0.133 | 0.031 | 0.184 |
|  | Hawaiian | 37 | 48 | 0.661 | 0.054 | 0.081 | 8021595.70 | 0.959 | 1.000 | 14388.168 | 0.854 | 1.000 | 131.969 | 0.596 | 1.000 | -0.001 | 0.446 | 1.000 | -0.010 | 0.665 | 1.000 |
|  | Iroquois | 70 | 15 | 0.097 | 0.468 | 0.007 | 4216334.11 | 0.032 | 0.190 | 8460.864 | 0.484 | 1.000 | 74.751 | 0.005 | 0.030 | 0.028 | 0.248 | 1.000 | -0.125 | 0.997 | 1.000 |
|  | Omaha | 84 | 1 | 2.860 | 0.230 | 0.700 | 430380.25 | 0.376 | 1.000 | 702.380 | 0.377 | 1.000 | 6.328 | 0.867 | 1.000 | 0.044 | 0.171 | 0.975 | -0.014 | 0.626 | 1.000 |
|  | Sudanese | 84 | 1 | -2.063 | 0.714 | 0.171 | 498098.38 | 0.260 | 1.000 | 709.856 | 0.281 | 1.000 | 6.220 | 0.826 | 1.000 | 0.067 | 0.118 | 0.710 | -0.035 | 0.674 | 1.000 |
| Bantu | Crow | 65 | 4 | 1.105 | 0.129 | 0.525 | 348598.29 | 0.887 | 1.000 | 1823.446 | 0.857 | 1.000 | 15359.566 | 0.627 | 1.000 | -0.011 | 0.512 | 1.000 | -0.063 | 0.670 | 1.000 |
|  | Descriptive | 68 | 1 | 2.941 | 0.066 | 0.881 | 93014.64 | 0.783 | 1.000 | 477.297 | 0.900 | 1.000 | 5417.879 | 0.233 | 1.000 | -0.031 | 0.603 | 1.000 | 0.136 | 0.077 | 0.537 |
|  | Hawaiian | 60 | 9 | 0.405 | 0.297 | 0.083 | 792380.62 | 0.524 | 1.000 | 3835.774 | 0.496 | 1.000 | 35032.624 | 0.542 | 1.000 | 0.031 | 0.284 | 1.000 | 0.040 | 0.288 | 1.000 |
|  | Iroquois | 25 | 44 | 0.166 | 0.362 | 0.008 | 1636008.46 | 0.023 | 0.159 | 7853.277 | 0.007 | 0.046 | 71119.715 | 0.151 | 0.892 | 0.105 | 0.005 | 0.038 | 0.051 | 0.138 | 0.863 |
|  | Mixed | 68 | 1 | -2.432 | 0.834 | 0.040 | 116322.82 | 0.320 | 1.000 | 500.446 | 0.290 | 1.000 | 3836.258 | 0.916 | 1.000 | 0.086 | 0.121 | 0.844 | -0.067 | 0.854 | 1.000 |
|  | Omaha | 61 | 8 | 0.767 | 0.140 | 0.274 | 733279.95 | 0.393 | 1.000 | 3484.320 | 0.309 | 1.000 | 32352.914 | 0.396 | 1.000 | 0.051 | 0.197 | 1.000 | 0.067 | 0.218 | 1.000 |
|  | Sudanese | 67 | 2 | 1.051 | 0.287 | 0.459 | 169263.56 | 0.718 | 1.000 | 929.736 | 0.623 | 1.000 | 10075.747 | 0.223 | 1.000 | -0.066 | 0.840 | 1.000 | 0.148 | 0.083 | 0.580 |
| Uto-Aztecan | Crow | 21 | 1 | 0.243 | 0.419 | 0.462 | 13195.12 | 0.365 | 1.000 | 132.056 | 0.998 | 1.000 | 0.025 | 0.659 | 0.990 | -0.022 | 0.484 | 1.000 | 0.060 | 0.341 | 0.935 |
|  | Eskimo | 21 | 1 | 3.253 | 0.216 | 0.679 | 14128.24 | 0.818 | 1.000 | 133.048 | 0.909 | 1.000 | 0.026 | 0.504 | 0.974 | -0.003 | 0.355 | 1.000 | 0.087 | 0.255 | 0.827 |
|  | Hawaiian | 6 | 16 | -0.600 | 0.798 | 0.004 | 70096.07 | 0.259 | 0.980 | 606.534 | 0.596 | 1.000 | 0.114 | 0.136 | 0.545 | -0.035 | 0.604 | 1.000 | 0.145 | 0.073 | 0.291 |
|  | Iroquois | 18 | 4 | -2.343 | 0.985 | 0.000 | 54649.14 | 0.881 | 1.000 | 457.499 | 0.973 | 1.000 | 0.086 | 0.235 | 0.840 | 0.001 | 0.438 | 1.000 | 0.134 | 0.120 | 0.479 |

# Ancestral state

## MCMC review

Ancestral state inference allows us to estimate the probability of a particular terminology at the root of each language phylogeny, as well as estimating the patterns of change between each state. We perform Bayesian reversible-jump MCMC ancestral state inference kinship terminologies using BayesTraits V 3.0 Multistate (Pagel and Meade 2017). Multistate uses a posterior of phylogenies to estimate the probability of each terminology present in the taxa at the phylogeny root, and an estimation of the rate (Q) matrix. The reversible-jump approach searches the model space for an optimal solution by dynamically setting some rate parameters (i.e. transitions from one state to another) to zero. This results in searching the model space where we are confident transition rates are non-zero. The Q matrix shows the likelihood of changing from any state to any other. MCMC chains were run for 10^9^ iterations, sampling every 50,000 iterations with a burn-in of 10,000 iterations, to give a posterior sample of 20,000. Each analysis ran three times to test consistent MCMC convergence using the Gelman-Rubin diagnostic (Gelman and Rubin 1992). Due to uncertainty in the inference of the Bantu ancestral state, each terminology present in the phylogeny was ‘fossilized’ as the root to estimate likelihoods. Effectively, this forces the algorithm to build a model given a particular ancestral state and given the contemporary data. We can then compare the model fit across each constrained model. We calculate pairwise Bayes factors (BF) to assess the evidence for each response. BF < 3 indicates weak evidence, >3 positive evidence, and >10 very strong evidence. This is shown in section 3.1.1.

Table S5: Three MCMC chains for the Austronesian language family. The base probability for any type is 0.167.

|  | Lh | Crow | Eskimo | Hawaiian | Iroquois | Omaha | Sudanese |
| --- | --- | --- | --- | --- | --- | --- | --- |
| 1 | -95.053 | 0.029 | 0.846 | 0.011 | 0.015 | 0.042 | 0.057 |
| 2 | -95.037 | 0.029 | 0.846 | 0.010 | 0.015 | 0.042 | 0.058 |
| 3 | -95.042 | 0.029 | 0.848 | 0.010 | 0.014 | 0.041 | 0.057 |
| Mean | -95.044 | 0.029 | 0.847 | 0.010 | 0.015 | 0.042 | 0.057 |

Table S6: Three MCMC chains for the Bantu language family. The base probability for any type is 0.143.

|  | Lh | Crow | Descriptive | Hawaiian | Iroquois | Omaha | Sudanese | Mixed |
| --- | --- | --- | --- | --- | --- | --- | --- | --- |
| 1 | -81.151 | 0.089 | 0.078 | 0.157 | 0.393 | 0.101 | 0.082 | 0.099 |
| 2 | -81.134 | 0.090 | 0.078 | 0.156 | 0.395 | 0.102 | 0.081 | 0.097 |
| 3 | -81.141 | 0.089 | 0.078 | 0.156 | 0.397 | 0.101 | 0.081 | 0.098 |
| Mean | -81.142 | 0.089 | 0.078 | 0.156 | 0.395 | 0.101 | 0.081 | 0.098 |

Table S7: Three MCMC chains for the Uto-Aztecan language family. The base probability for any type is 0.25.

|  | Lh | Crow | Eskimo | Hawaiian | Iroquois |
| --- | --- | --- | --- | --- | --- |
| 1 | -19.279 | 0.232 | 0.226 | 0.310 | 0.232 |
| 2 | -19.291 | 0.232 | 0.224 | 0.312 | 0.232 |
| 3 | -19.282 | 0.232 | 0.225 | 0.311 | 0.232 |
| Mean | -19.284 | 0.232 | 0.225 | 0.311 | 0.232 |

Table S8: Gelman-Rubin tests of MCMC convergence for each language family

|  | Point est. | Upper C.I. |
| --- | --- | --- |
| austronesian | 1 | 1 |
| bantu | 1 | 1 |
| Uto-Aztecan | 1 | 1 |

### Fossilized Bantu

#### MCMC Review

We fossilize the ancestral state for each possible terminology within Bantu in an attempt to determine the most plausible ancestral state. Below are the marginal log-likelihoods for these models and the result with comparisons to the most likely ancestral state, Iroquoian.

Table S9: Marginal log-likelihood for each fossilized terminology in Bantu

| terminology | MLL |
| --- | --- |
| Crow | -91.683 |
| Descriptive | -90.914 |
| Hawaiian | -91.450 |
| Iroquois | -90.297 |
| Mixed | -91.819 |
| Omaha | -90.805 |
| Sudanese | -92.595 |

####

#### Bayes factor model comparison

Bayes factor calculations show that there is some evidence for an Iroquoian root over a Hawaiian root (BF = 2.306). The table below shows comparisons to the fossilized Iroquois root to all other fossilized roots.

Table S10: Pairwise Bayes factor between Iroquois and all other possible states.

| terminology | Bayes’ Factor |
| --- | --- |
| Crow | 2.772 |
| Descriptive | 1.235 |
| Hawaiian | 2.306 |
| Iroquois | 0.000 |
| Mixed | 3.043 |
| Omaha | 1.017 |
| Sudanese | 4.595 |

### Fossilized Uto-Aztecan

#### MCMC Review

As with Bantu, we fossilize the ancestral state for each possible terminology within Uto-Aztecan to determine the most plausible ancestral state. Below are the Bayes Factor comparisons between the most likely ancestral state & all other possible states. There is no preference for a particular ancestral state.

Table S12: Pairwise Bayes factor between Hawaiian and all other possible states.

| terminology | Bayes’ Factor |
| --- | --- |
| Crow | -0.103 |
| Eskimo | 0.058 |
| Hawaiian | 0.000 |
| Iroquois | 0.852 |

##

## PMP in Austronesia

Here we reconstruct the PMP state in Austronesian using the RecNode command in BayesTraits. All other settings remain the same as the Austronesian model.

Table S13: Ancestral state for PMP

|  | Probability |
| --- | --- |
| RecNode P(1) | 0.020 |
| RecNode P(3) | 0.897 |
| RecNode P(4) | 0.004 |
| RecNode P(5) | 0.006 |
| RecNode P(6) | 0.026 |
| RecNode P(7) | 0.046 |

##

## Transition rates

RJ ancestral state analysis also estimates the rate of transition between kinship terminological types. The model constrains the number of parameters estimated, forcing some parameters to be zero, and then estimates and appropriate number of parameters for accurately represent the model. By looking at which parameters are set to zero across the MCMC chain, and comparing that with the number of times we would expect a transition to be estimated under the priors of the model we can see which transition rates the model believes to be important (Currie et al., 2010). We assume that if the posterior-to-prior odds are less than one, we have no evidence to support a particular transition being zero (*italicized rows have prior-posterior odds less than 1*).

Table S14: Transition rates between kin terminologies in the Austronesian language family.

| transition | parameterized | zero | percentage | posterior-prior  odds |
| --- | --- | --- | --- | --- |
| *Crow -> Hawaiian* | *9160* | *840* | *0.916* | *0.349* |
| *Eskimo -> Hawaiian* | *9080* | *920* | *0.908* | *0.386* |
| *Hawaiian -> Iroquois* | *8921* | *1079* | *0.892* | *0.461* |
| Omaha -> Hawaiian | 7260 | 2740 | 0.726 | 1.438 |
| Sudanese -> Hawaiian | 7114 | 2886 | 0.711 | 1.545 |
| Iroquois -> Hawaiian | 6993 | 3007 | 0.699 | 1.638 |
| Omaha -> Crow | 6919 | 3081 | 0.692 | 1.696 |
| Crow -> Iroquois | 6792 | 3208 | 0.679 | 1.799 |
| Omaha -> Sudanese | 6561 | 3439 | 0.656 | 1.997 |
| Crow -> Omaha | 6499 | 3501 | 0.650 | 2.052 |
| Omaha -> Iroquois | 6436 | 3564 | 0.644 | 2.110 |
| Sudanese -> Crow | 6305 | 3695 | 0.631 | 2.233 |
| Iroquois -> Crow | 6270 | 3730 | 0.627 | 2.266 |
| Sudanese -> Omaha | 6128 | 3872 | 0.613 | 2.407 |
| Crow -> Sudanese | 5964 | 4036 | 0.596 | 2.578 |
| Sudanese -> Iroquois | 5875 | 4125 | 0.588 | 2.675 |
| Hawaiian -> Crow | 5622 | 4378 | 0.562 | 2.967 |
| Sudanese -> Eskimo | 4980 | 5020 | 0.498 | 3.840 |
| Omaha -> Eskimo | 4876 | 5124 | 0.488 | 4.003 |
| Iroquois -> Omaha | 4191 | 5809 | 0.419 | 5.280 |
| Crow -> Eskimo | 3699 | 6301 | 0.370 | 6.489 |
| Iroquois -> Sudanese | 3229 | 6771 | 0.323 | 7.988 |
| Eskimo -> Sudanese | 2182 | 7818 | 0.218 | 13.649 |
| Hawaiian -> Omaha | 1851 | 8149 | 0.185 | 16.771 |
| Hawaiian -> Sudanese | 1279 | 8721 | 0.128 | 25.976 |
| Eskimo -> Crow | 1005 | 8995 | 0.101 | 34.096 |
| Eskimo -> Omaha | 953 | 9047 | 0.095 | 36.165 |
| Iroquois -> Eskimo | 846 | 9154 | 0.085 | 41.221 |
| Eskimo -> Iroquois | 410 | 9590 | 0.041 | 89.106 |
| Hawaiian -> Eskimo | 255 | 9745 | 0.025 | 145.584 |

Table S15: Transition rates between kin terminologies in the Bantu language family.

| transition | parameterized | zero | percentage | posterior-prior  odds |
| --- | --- | --- | --- | --- |
| *Iroquois -> Omaha* | *9163* | *837* | *0.916* | *0.420* |
| Iroquois -> Hawaiian | 8137 | 1863 | 0.814 | 1.053 |
| Omaha -> Descriptive | 7956 | 2044 | 0.796 | 1.182 |
| Crow -> Sudanese | 7449 | 2551 | 0.745 | 1.575 |
| Omaha -> Iroquois | 7339 | 2661 | 0.734 | 1.668 |
| Sudanese -> Crow | 6938 | 3062 | 0.694 | 2.030 |
| Crow -> Hawaiian | 6928 | 3072 | 0.693 | 2.040 |
| Hawaiian -> Crow | 6716 | 3284 | 0.672 | 2.249 |
| Omaha -> Hawaiian | 6447 | 3553 | 0.645 | 2.535 |
| Crow -> Iroquois | 6390 | 3610 | 0.639 | 2.599 |
| Sudanese -> Hawaiian | 6330 | 3670 | 0.633 | 2.667 |
| Omaha -> Crow | 6181 | 3819 | 0.618 | 2.842 |
| Descriptive -> Omaha | 6119 | 3881 | 0.612 | 2.917 |
| Omaha -> Sudanese | 5996 | 4004 | 0.600 | 3.072 |
| Hawaiian -> Iroquois | 5875 | 4125 | 0.588 | 3.229 |
| Descriptive -> Hawaiian | 5834 | 4166 | 0.583 | 3.285 |
| Sudanese -> Omaha | 5834 | 4166 | 0.583 | 3.285 |
| Mixed -> Hawaiian | 5722 | 4278 | 0.572 | 3.439 |
| Sudanese -> Iroquois | 5716 | 4284 | 0.572 | 3.447 |
| Descriptive -> Crow | 5576 | 4424 | 0.558 | 3.649 |
| Crow -> Omaha | 5546 | 4454 | 0.555 | 3.694 |
| Mixed -> Omaha | 5517 | 4483 | 0.552 | 3.738 |
| Mixed -> Crow | 5441 | 4559 | 0.544 | 3.854 |
| Descriptive -> Mixed | 5430 | 4570 | 0.543 | 3.871 |
| Sudanese -> Mixed | 5424 | 4576 | 0.542 | 3.880 |
| Omaha -> Mixed | 5417 | 4583 | 0.542 | 3.891 |
| Mixed -> Iroquois | 5411 | 4589 | 0.541 | 3.901 |
| Crow -> Mixed | 5362 | 4638 | 0.536 | 3.979 |
| Descriptive -> Sudanese | 5292 | 4708 | 0.529 | 4.092 |
| Descriptive -> Iroquois | 5280 | 4720 | 0.528 | 4.112 |
| Sudanese -> Descriptive | 5222 | 4778 | 0.522 | 4.209 |
| Mixed -> Sudanese | 5086 | 4914 | 0.509 | 4.444 |
| Iroquois -> Crow | 5075 | 4925 | 0.508 | 4.464 |
| Mixed -> Descriptive | 4959 | 5041 | 0.496 | 4.676 |
| Crow -> Descriptive | 4762 | 5238 | 0.476 | 5.059 |
| Hawaiian -> Omaha | 4197 | 5803 | 0.420 | 6.360 |
| Hawaiian -> Sudanese | 4080 | 5920 | 0.408 | 6.674 |
| Hawaiian -> Mixed | 3473 | 6527 | 0.347 | 8.644 |
| Hawaiian -> Descriptive | 2656 | 7344 | 0.266 | 12.718 |
| Iroquois -> Sudanese | 1774 | 8226 | 0.177 | 21.328 |
| Iroquois -> Mixed | 379 | 9621 | 0.038 | 116.762 |
| Iroquois -> Descriptive | 208 | 9792 | 0.021 | 216.534 |

Table S16: Transition rates between kin terminologies in the Uto-Aztecan language family.

| transition | parameterized | zero | percentage | posterior-prior  odds |
| --- | --- | --- | --- | --- |
| *Iroquois -> Hawaiian* | *9511* | *489* | *0.951* | *0.123* |
| *Crow -> Hawaiian* | *8658* | *1342* | *0.866* | *0.371* |
| *Eskimo -> Hawaiian* | *8393* | *1607* | *0.839* | *0.459* |
| *Hawaiian -> Iroquois* | *8036* | *1964* | *0.804* | *0.585* |
| *Eskimo -> Iroquois* | *7254* | *2746* | *0.725* | *0.907* |
| *Crow -> Iroquois* | *7220* | *2780* | *0.722* | *0.922* |
| Crow -> Eskimo | 6975 | 3025 | 0.698 | 1.039 |
| Iroquois -> Eskimo | 6877 | 3123 | 0.688 | 1.088 |
| Eskimo -> Crow | 6736 | 3264 | 0.674 | 1.160 |
| Iroquois -> Crow | 6240 | 3760 | 0.624 | 1.443 |
| Hawaiian -> Crow | 1380 | 8620 | 0.138 | 14.960 |
| Hawaiian -> Eskimo | 1336 | 8664 | 0.134 | 15.531 |

##

## Guillon and Mace comparison

Previous research performed by Guillon and Mace in, using similar methods, found support for a Hawaiian root, with some evidence of an Iroquoian root within the Bantu language family. The numerical results for this analysis are not available in the paper. This is the inverse of our result, which found most support for an Iroquoian root, with some support for Hawaiian. This discrepancy is concerning considering the terminological data for both analyses come from D-PLACE. The primary difference between approaches is that here we use Grollemund et al. (2015) more recently developed phylogeny (against Currie et al. (2014)), and secondarily, Guillon and Mace include missing data, where we exclude it. We re-analysed our data including missing data and found no change in our previous conclusions. Suggesting that the improved phylogeny is driving the change in results.

Table S17: Ancestral state of Bantu with missing data included

|  | probability |
| --- | --- |
| Crow | 0.124 |
| Descriptive | 0.117 |
| Hawaiian | 0.139 |
| Iroquois | 0.259 |
| Omaha | 0.124 |
| Sudanese | 0.119 |
| Mixed | 0.118 |

# Co-evolution tests

## MCMC settings

All co-evolutionary analyses was performed in BayesTraits v3.1 using Discrete models (M. Pagel & Meade, 2017). For all models we used a Reversible Jump MCMC approach and tested an independent and dependent model of evolution. In an independent model, a trait can change, regardless of the state of the other trait. In a dependent model, a trait change is dependent on the state of the second trait. If co-evolution has occurred, the data should fit a dependent model better than it fits an independent model. We test this using Bayes Factors and the same guides as described previously in the paper and SM. Analyses were run between 10,010,000 and 65,000,000 iterations, with burn-ins ranging between 10,000 and 55,000,000, and sampling every 1000 iterations. The results are a posterior of 10,000 iterations, approximately 10 iterations per tree per hypotheses. A stepping stone sampler was used to estimate the marginal likelihood. We used 100 stones sampled every 1,000 iterations. For details of the prior and other settings for each hypothesis, see the table below.

Table S18: MCMC settings for both dependent and independent analyses

| hypotheses | exp. mean | scaled branch length | iterations | sampling | burn-in | posterior size |
| --- | --- | --- | --- | --- | --- | --- |
| austronesian-crow-high.polygyny | 10 | Yes | 10050000 | 1000 | 50000 | 10000 |
| austronesian-crow-matri.anvunclocalresidence | 10 | Yes | 10050000 | 1000 | 50000 | 10000 |
| austronesian-crow-matrilineal | 10 | Yes | 10050000 | 1000 | 50000 | 10000 |
| austronesian-crow-polygyny | 10 | Yes | 10050000 | 1000 | 50000 | 10000 |
| austronesian-crow-matrilocal | 10 | Yes | 10010000 | 1000 | 10000 | 10000 |
| austronesian-crow-matrilocal.strict | 10 | Yes | 10010000 | 1000 | 10000 | 10000 |
| austronesian-crow-uni.localresidence | 10 | Yes | 10010000 | 1000 | 10000 | 10000 |
| austronesian-eskimo-absenceofcousinmarriage.permitted | 10 | Yes | 65000000 | 1000 | 55000000 | 10000 |
| austronesian-eskimo-absenceofcousinmarriage.preference | 10 | Yes | 65000000 | 1000 | 55000000 | 10000 |
| austronesian-eskimo-bi.linealdescent | 10 | Yes | 10010000 | 1000 | 10000 | 10000 |
| austronesian-eskimo-monogamy | 10 | Yes | 10050000 | 1000 | 50000 | 10000 |
| austronesian-eskimo-neo.localresidence | 10 | Yes | 65000000 | 1000 | 55000000 | 10000 |
| austronesian-eskimo-nuclear.families | 10 | Yes | 10010000 | 1000 | 10000 | 10000 |
| austronesian-eskimo-uni.linealdescent | 10 | Yes | 10010000 | 1000 | 10000 | 10000 |
| austronesian-hawaiian-absenceofcousinmarriage.permitted | 10 | Yes | 10050000 | 1000 | 50000 | 10000 |
| austronesian-hawaiian-absenceofcousinmarriage.preference | 10 | Yes | 10010000 | 1000 | 10000 | 10000 |
| austronesian-hawaiian-bi.linealdescent | 10 | Yes | 10010000 | 1000 | 10000 | 10000 |
| austronesian-hawaiian-bi.localextendedfamily | 10 | Yes | 10010000 | 1000 | 10000 | 10000 |
| austronesian-hawaiian-bi.localresidence | 10 | Yes | 10050000 | 1000 | 50000 | 10000 |
| austronesian-iroquois-cross.cousinmarriage.permitted | 10 | Yes | 10010000 | 1000 | 10000 | 10000 |
| austronesian-iroquois-cross.cousinmarriage.preferred | 10 | Yes | 10010000 | 1000 | 10000 | 10000 |
| austronesian-iroquois-exogamy.unilineal.descent | 10 | Yes | 10010000 | 1000 | 10000 | 10000 |
| austronesian-iroquois-high.polygyny | 10 | Yes | 10010000 | 1000 | 10000 | 10000 |
| austronesian-iroquois-matri.anvunclocalresidence | 10 | Yes | 10010000 | 1000 | 10000 | 10000 |
| austronesian-iroquois-polygyny | 10 | Yes | 10010000 | 1000 | 10000 | 10000 |
| austronesian-iroquois-uni.linealdescent | 10 | Yes | 10010000 | 1000 | 10000 | 10000 |
| austronesian-iroquois-uni.localresidence | 10 | Yes | 10050000 | 1000 | 50000 | 10000 |
| bantu-crow-matri.anvunclocalresidence | 10 | Yes | 10010000 | 1000 | 10000 | 10000 |
| bantu-crow-matrilineal | 10 | Yes | 10010000 | 1000 | 10000 | 10000 |
| bantu-crow-matrilocal | 10 | Yes | 10050000 | 1000 | 50000 | 10000 |
| bantu-crow-matrilocal.strict | 10 | Yes | 10050000 | 1000 | 50000 | 10000 |
| bantu-crow-uni.localresidence | 10 | Yes | 10010000 | 1000 | 10000 | 10000 |
| bantu-hawaiian-absenceofcousinmarriage.permitted | 10 | Yes | 10010000 | 1000 | 10000 | 10000 |
| bantu-hawaiian-absenceofcousinmarriage.preference | 10 | Yes | 10010000 | 1000 | 10000 | 10000 |
| bantu-hawaiian-bi.linealdescent | 10 | Yes | 10010000 | 1000 | 10000 | 10000 |
| bantu-hawaiian-bi.localextendedfamily | 10 | Yes | 10010000 | 1000 | 10000 | 10000 |
| bantu-hawaiian-bi.localresidence | 10 | Yes | 10010000 | 1000 | 10000 | 10000 |
| bantu-iroquois-cross.cousinmarriage.permitted | 10 | Yes | 10010000 | 1000 | 10000 | 10000 |
| bantu-iroquois-cross.cousinmarriage.preferred | 10 | Yes | 10010000 | 1000 | 10000 | 10000 |
| bantu-iroquois-exogamy.unilineal.descent | 10 | Yes | 10010000 | 1000 | 10000 | 10000 |
| bantu-iroquois-matri.anvunclocalresidence | 10 | Yes | 10010000 | 1000 | 10000 | 10000 |
| bantu-iroquois-uni.linealdescent | 10 | Yes | 10010000 | 1000 | 10000 | 10000 |
| bantu-iroquois-uni.localresidence | 10 | Yes | 10010000 | 1000 | 10000 | 10000 |
| bantu-omaha-matri.anvunclocalresidence | 10 | Yes | 10010000 | 1000 | 10000 | 10000 |
| bantu-omaha-patrilineal | 10 | Yes | 10010000 | 1000 | 10000 | 10000 |
| bantu-omaha-patrilocal | 10 | Yes | 10010000 | 1000 | 10000 | 10000 |
| bantu-omaha-patrilocal.strict | 10 | Yes | 10010000 | 1000 | 10000 | 10000 |
| bantu-omaha-uni.localresidence | 10 | Yes | 10010000 | 1000 | 10000 | 10000 |
| uto-hawaiian-absenceofcousinmarriage.permitted | 10 | Yes | 10010000 | 1000 | 10000 | 10000 |
| uto-hawaiian-absenceofcousinmarriage.preference | 10 | Yes | 10010000 | 1000 | 10000 | 10000 |
| uto-hawaiian-bi.linealdescent | 10 | Yes | 10010000 | 1000 | 10000 | 10000 |
| uto-hawaiian-bi.localextendedfamily | 10 | Yes | 10010000 | 1000 | 10000 | 10000 |
| uto-hawaiian-bi.localresidence | 10 | Yes | 10010000 | 1000 | 10000 | 10000 |
| uto-iroquois-cross.cousinmarriage.permitted | 10 | Yes | 10010000 | 1000 | 10000 | 10000 |
| uto-iroquois-cross.cousinmarriage.preferred | 10 | Yes | 10010000 | 1000 | 10000 | 10000 |
| uto-iroquois-exogamy.unilineal.descent | 10 | Yes | 10010000 | 1000 | 10000 | 10000 |
| uto-iroquois-high.polygyny | 10 | Yes | 10010000 | 1000 | 10000 | 10000 |
| uto-iroquois-matri.anvunclocalresidence | 10 | Yes | 10010000 | 1000 | 10000 | 10000 |
| uto-iroquois-uni.linealdescent | 10 | Yes | 10010000 | 1000 | 10000 | 10000 |
| uto-iroquois-uni.localresidence | 10 | Yes | 10010000 | 1000 | 10000 | 10000 |

##

## MCMC review and Bayes Factors

Here we show the results of MCMC chains and respective tests. The first table for each hypothesis is a review of the MCMC chains. Each row shows the marginal log-likelihood and averaged transition rates for the dependent and independent models for a single chain. There are three MCMC chains run for each hypothesis to ensure the model is reaching the same conclusion each time. The penultimate row shows the mean across the three runs. The final row tests which model was more appropriate for the data. We calculate a Bayes-factor, which compares the log marginal likelihood between the dependent and independent models, as explained in Pagel & Meade (2006). A Bayes Factor greater than ten indicates strong support, greater than three positive support and less than three no support.

A second table shows the results of a Gelman-Rubin diagnostic testing, to test for any significant differences between chains (Gelman & Rubin, 1992). A value of 1 indicates no difference, and a rule of thumb suggests point-estimates of less than 1.1 indicate negligible differences between chains.

Some extra analyses are also shown here that are not seen in table 1. These are matrilocal and patrilocal hypotheses and matrilocal.strict and patrilocal.strict. The strict hypothesis excludes anything, but societies classified as patrilocal or matrilocal. In the main text we discuss the differences between including virilocality and uxorilocality under each of these hypotheses, there are represented in the ‘not strict’ hypothesis.

Table S19: Austronesian : Crow <-> High.polygyny

| run | MLL | q12 | q13 | q21 | q24 | q31 | q34 | q42 | q43 | MLL | α1 | β1 | α2 | β2 |
| --- | --- | --- | --- | --- | --- | --- | --- | --- | --- | --- | --- | --- | --- | --- |
| 1 | -58.486 | 2.537 | 1.219 | 17.085 | 2.878 | 23.205 | 4.089 | 7.454 | 6.586 | -59.016 | 1.68 | 21.444 | 2.778 | 15.975 |
| 2 | -58.607 | 2.521 | 1.259 | 16.807 | 2.979 | 23.77 | 4.074 | 7.654 | 6.568 | -58.942 | 1.704 | 21.656 | 2.777 | 15.799 |
| 3 | -58.54 | 2.546 | 1.236 | 17.188 | 2.985 | 23.439 | 4.042 | 7.67 | 6.556 | -59.1 | 1.688 | 21.731 | 2.827 | 16.154 |
| Mean | -58.544 | 2.535 | 1.238 | 17.027 | 2.947 | 23.471 | 4.068 | 7.593 | 6.57 | -59.019 | 1.691 | 21.61 | 2.794 | 15.976 |
| BF | 1.059 |  |  |  |  |  |  |  |  |  |  |  |  |  |

Gelman-Rubin MCMC chain diagnostic test

|  | Point est. | Upper C.I. |
| --- | --- | --- |
| Dependent | 1 | 1 |
| Independent | 1 | 1 |

Table S20: Austronesian : Crow <-> Matri.anvunclocalresidence

| run | MLL | q12 | q13 | q21 | q24 | q31 | q34 | q42 | q43 | MLL | α1 | β1 | α2 | β2 |
| --- | --- | --- | --- | --- | --- | --- | --- | --- | --- | --- | --- | --- | --- | --- |
| 1 | -54.688 | 0.997 | 0.367 | 6.689 | 8.886 | 12.439 | 10.346 | 17.49 | 4.623 | -59.875 | 1.604 | 21.99 | 1.166 | 5.64 |
| 2 | -54.6 | 1.007 | 0.348 | 6.647 | 8.906 | 12.272 | 9.917 | 17.536 | 4.619 | -60.003 | 1.622 | 22.299 | 1.165 | 5.674 |
| 3 | -54.71 | 0.991 | 0.366 | 6.628 | 8.98 | 12.309 | 10.322 | 17.464 | 4.705 | -59.84 | 1.609 | 21.951 | 1.165 | 5.626 |
| Mean | -54.666 | 0.998 | 0.36 | 6.655 | 8.924 | 12.34 | 10.195 | 17.497 | 4.649 | -59.906 | 1.612 | 22.08 | 1.165 | 5.647 |
| BF | 10.374 |  |  |  |  |  |  |  |  |  |  |  |  |  |

Gelman-Rubin MCMC chain diagnostic test

|  | Point est. | Upper C.I. |
| --- | --- | --- |
| Dependent | 1 | 1 |
| Independent | 1 | 1 |

Table S21: Austronesian : Crow <-> Matrilineal

| run | MLL | q12 | q13 | q21 | q24 | q31 | q34 | q42 | q43 | MLL | α1 | β1 | α2 | β2 |
| --- | --- | --- | --- | --- | --- | --- | --- | --- | --- | --- | --- | --- | --- | --- |
| 1 | -55.963 | 0.755 | 0.324 | 5.045 | 8.59 | 12.393 | 11.438 | 17.922 | 8.894 | -61.187 | 1.573 | 21.589 | 1.01 | 6.265 |
| 2 | -56.018 | 0.765 | 0.318 | 5.079 | 8.606 | 12.566 | 11.038 | 17.838 | 8.997 | -61.205 | 1.575 | 21.477 | 1 | 6.224 |
| 3 | -56.128 | 0.751 | 0.322 | 5.082 | 8.556 | 12.417 | 11.061 | 17.805 | 8.803 | -61.206 | 1.574 | 21.449 | 1.003 | 6.315 |
| Mean | -56.036 | 0.757 | 0.321 | 5.069 | 8.584 | 12.459 | 11.179 | 17.855 | 8.898 | -61.199 | 1.574 | 21.505 | 1.004 | 6.268 |
| BF | 10.45 |  |  |  |  |  |  |  |  |  |  |  |  |  |

Gelman-Rubin MCMC chain diagnostic test

|  | Point est. | Upper C.I. |
| --- | --- | --- |
| Dependent | 1 | 1 |
| Independent | 1 | 1 |

Table S22: Austronesian : Crow <-> Polygyny

| run | MLL | q12 | q13 | q21 | q24 | q31 | q34 | q42 | q43 | MLL | α1 | β1 | α2 | β2 |
| --- | --- | --- | --- | --- | --- | --- | --- | --- | --- | --- | --- | --- | --- | --- |
| 1 | -68.567 | 2.689 | 2.512 | 1.505 | 0.917 | 24.339 | 6.913 | 9.728 | 4.795 | -67.647 | 1.657 | 21.326 | 2.6 | 1.569 |
| 2 | -68.762 | 2.715 | 2.488 | 1.519 | 0.922 | 24.556 | 6.997 | 9.912 | 4.883 | -67.63 | 1.659 | 21.246 | 2.606 | 1.566 |
| 3 | -68.648 | 2.711 | 2.553 | 1.529 | 0.921 | 24.159 | 6.962 | 9.776 | 4.906 | -67.461 | 1.661 | 21.217 | 2.652 | 1.576 |
| Mean | -68.659 | 2.705 | 2.518 | 1.518 | 0.92 | 24.351 | 6.957 | 9.805 | 4.861 | -67.579 | 1.659 | 21.263 | 2.619 | 1.57 |
| BF | -1.839 |  |  |  |  |  |  |  |  |  |  |  |  |  |

Gelman-Rubin MCMC chain diagnostic test

|  | Point est. | Upper C.I. |
| --- | --- | --- |
| Dependent | 1 | 1.001 |
| Independent | 1 | 1.001 |

Table S23: Austronesian : Crow <-> Matrilocal

| run | MLL | q12 | q13 | q21 | q24 | q31 | q34 | q42 | q43 | MLL | α1 | β1 | α2 | β2 |
| --- | --- | --- | --- | --- | --- | --- | --- | --- | --- | --- | --- | --- | --- | --- |
| 1 | -52.623 | 0.781 | 0.535 | 6.674 | 7.172 | 9.728 | 4.808 | 19.083 | 6.265 | -53.849 | 1.553 | 20.979 | 0.787 | 5.359 |
| 2 | -52.695 | 0.766 | 0.551 | 6.503 | 7.071 | 9.989 | 4.887 | 18.722 | 6.404 | -53.885 | 1.554 | 21.072 | 0.777 | 5.377 |
| 3 | -52.637 | 0.781 | 0.555 | 6.751 | 7.007 | 9.774 | 5.053 | 18.934 | 6.29 | -53.889 | 1.545 | 21.074 | 0.781 | 5.354 |
| Mean | -52.652 | 0.776 | 0.547 | 6.643 | 7.083 | 9.83 | 4.916 | 18.913 | 6.32 | -53.874 | 1.551 | 21.042 | 0.782 | 5.363 |
| BF | 2.452 |  |  |  |  |  |  |  |  |  |  |  |  |  |

Gelman-Rubin MCMC chain diagnostic test

|  | Point est. | Upper C.I. |
| --- | --- | --- |
| Dependent | 1 | 1.001 |
| Independent | 1 | 1.000 |

Table S24: Austronesian : Crow <-> Matrilocal.strict

| run | MLL | q12 | q13 | q21 | q24 | q31 | q34 | q42 | q43 | MLL | α1 | β1 | α2 | β2 |
| --- | --- | --- | --- | --- | --- | --- | --- | --- | --- | --- | --- | --- | --- | --- |
| 1 | -46.668 | 0.472 | 0.528 | 5.731 | 8.376 | 9.268 | 4.732 | 18.061 | 5.755 | -48.368 | 1.524 | 20.319 | 0.5 | 4.531 |
| 2 | -46.868 | 0.473 | 0.539 | 5.714 | 8.355 | 9.402 | 4.752 | 18.237 | 5.754 | -48.467 | 1.48 | 20.299 | 0.506 | 4.565 |
| 3 | -46.751 | 0.474 | 0.528 | 5.738 | 8.262 | 9.218 | 4.623 | 18.336 | 5.835 | -48.524 | 1.51 | 20.168 | 0.501 | 4.466 |
| Mean | -46.762 | 0.473 | 0.532 | 5.728 | 8.331 | 9.296 | 4.702 | 18.211 | 5.781 | -48.453 | 1.505 | 20.262 | 0.502 | 4.521 |
| BF | 3.401 |  |  |  |  |  |  |  |  |  |  |  |  |  |

Gelman-Rubin MCMC chain diagnostic test

|  | Point est. | Upper C.I. |
| --- | --- | --- |
| Dependent | 1 | 1 |
| Independent | 1 | 1 |

Table S25: Austronesian : Crow <-> Uni.localresidence

| run | MLL | q12 | q13 | q21 | q24 | q31 | q34 | q42 | q43 | MLL | α1 | β1 | α2 | β2 |
| --- | --- | --- | --- | --- | --- | --- | --- | --- | --- | --- | --- | --- | --- | --- |
| 1 | -72.461 | 1.442 | 0.316 | 2.925 | 4.261 | 14.85 | 10.992 | 20.857 | 2.46 | -74.574 | 1.325 | 17.603 | 1.477 | 2.675 |
| 2 | -72.68 | 1.466 | 0.318 | 2.9 | 4.179 | 15.043 | 10.985 | 20.603 | 2.418 | -74.309 | 1.311 | 17.577 | 1.496 | 2.682 |
| 3 | -72.343 | 1.459 | 0.322 | 2.924 | 4.109 | 14.996 | 11.26 | 20.652 | 2.414 | -74.434 | 1.312 | 17.239 | 1.493 | 2.693 |
| Mean | -72.495 | 1.456 | 0.319 | 2.916 | 4.183 | 14.963 | 11.079 | 20.704 | 2.431 | -74.439 | 1.316 | 17.473 | 1.489 | 2.683 |
| BF | 4.228 |  |  |  |  |  |  |  |  |  |  |  |  |  |

Gelman-Rubin MCMC chain diagnostic test

|  | Point est. | Upper C.I. |
| --- | --- | --- |
| Dependent | 1 | 1.001 |
| Independent | 1 | 1.000 |

Table S26: Austronesian : Eskimo <-> Absenceofcousinmarriage.permitted

| run | MLL | q12 | q13 | q21 | q24 | q31 | q34 | q42 | q43 | MLL | α1 | β1 | α2 | β2 |
| --- | --- | --- | --- | --- | --- | --- | --- | --- | --- | --- | --- | --- | --- | --- |
| 1 | -79.717 | 14.908 | 1.986 | 3.834 | 0.336 | 5.256 | 7.162 | 3.325 | 5.801 | -79.556 | 0.252 | 3.432 | 14.372 | 5.077 |
| 2 | -79.605 | 15.275 | 1.965 | 3.9 | 0.329 | 5.323 | 7.195 | 3.27 | 5.932 | -79.494 | 0.251 | 3.422 | 14.448 | 5.046 |
| 3 | -79.732 | 14.862 | 1.948 | 3.843 | 0.349 | 5.299 | 7.131 | 3.281 | 5.823 | -79.556 | 0.262 | 3.431 | 14.551 | 5.053 |
| Mean | -79.685 | 15.015 | 1.966 | 3.859 | 0.338 | 5.293 | 7.163 | 3.292 | 5.852 | -79.535 | 0.255 | 3.428 | 14.457 | 5.059 |
| BF | -0.323 |  |  |  |  |  |  |  |  |  |  |  |  |  |

Gelman-Rubin MCMC chain diagnostic test

|  | Point est. | Upper C.I. |
| --- | --- | --- |
| Dependent | 1 | 1.000 |
| Independent | 1 | 1.002 |

Table S27: Austronesian : Eskimo <-> Absenceofcousinmarriage.preference

| run | MLL | q12 | q13 | q21 | q24 | q31 | q34 | q42 | q43 | MLL | α1 | β1 | α2 | β2 |
| --- | --- | --- | --- | --- | --- | --- | --- | --- | --- | --- | --- | --- | --- | --- |
| 1 | -70.961 | 4.325 | 0.376 | 18.722 | 1.117 | 3.242 | 0.926 | 11.685 | 13.576 | -69.75 | 0.252 | 3.443 | 3.733 | 20.012 |
| 2 | -70.936 | 4.356 | 0.38 | 18.774 | 1.126 | 3.254 | 0.945 | 11.431 | 13.434 | -69.81 | 0.251 | 3.451 | 3.748 | 19.94 |
| 3 | -70.597 | 4.356 | 0.382 | 18.867 | 1.106 | 3.226 | 0.91 | 11.764 | 13.332 | -69.799 | 0.26 | 3.458 | 3.689 | 19.721 |
| Mean | -70.831 | 4.346 | 0.379 | 18.788 | 1.116 | 3.241 | 0.927 | 11.627 | 13.447 | -69.786 | 0.254 | 3.451 | 3.723 | 19.891 |
| BF | -2.423 |  |  |  |  |  |  |  |  |  |  |  |  |  |

Gelman-Rubin MCMC chain diagnostic test

|  | Point est. | Upper C.I. |
| --- | --- | --- |
| Dependent | 1.001 | 1.002 |
| Independent | 1.000 | 1.000 |

Table S28: Austronesian : Eskimo <-> Bi.linealdescent

| run | MLL | q12 | q13 | q21 | q24 | q31 | q34 | q42 | q43 | MLL | α1 | β1 | α2 | β2 |
| --- | --- | --- | --- | --- | --- | --- | --- | --- | --- | --- | --- | --- | --- | --- |
| 1 | -92.099 | 4.625 | 0.685 | 5.322 | 0.5 | 3.896 | 1.499 | 5.741 | 9.434 | -91.281 | 0.165 | 3.213 | 2.651 | 4.338 |
| 2 | -92.021 | 4.655 | 0.661 | 5.433 | 0.506 | 3.879 | 1.526 | 5.903 | 9.614 | -91.134 | 0.167 | 3.199 | 2.645 | 4.319 |
| 3 | -91.91 | 4.65 | 0.666 | 5.415 | 0.495 | 3.879 | 1.521 | 5.832 | 9.371 | -91.503 | 0.161 | 3.183 | 2.619 | 4.302 |
| Mean | -92.01 | 4.643 | 0.671 | 5.39 | 0.5 | 3.885 | 1.515 | 5.825 | 9.473 | -91.306 | 0.164 | 3.198 | 2.638 | 4.32 |
| BF | -1.634 |  |  |  |  |  |  |  |  |  |  |  |  |  |

Gelman-Rubin MCMC chain diagnostic test

|  | Point est. | Upper C.I. |
| --- | --- | --- |
| Dependent | 1 | 1.000 |
| Independent | 1 | 1.001 |

Table S29: Austronesian : Eskimo <-> Monogamy

| run | MLL | q12 | q13 | q21 | q24 | q31 | q34 | q42 | q43 | MLL | α1 | β1 | α2 | β2 |
| --- | --- | --- | --- | --- | --- | --- | --- | --- | --- | --- | --- | --- | --- | --- |
| 1 | -70.882 | 0.964 | 0.331 | 3.596 | 5.71 | 4.521 | 9.368 | 5.121 | 5.901 | -74.566 | 0.188 | 3.145 | 1.265 | 2.361 |
| 2 | -70.993 | 0.956 | 0.322 | 3.639 | 5.569 | 4.46 | 9.625 | 5.075 | 6.011 | -74.593 | 0.187 | 3.174 | 1.269 | 2.382 |
| 3 | -71.014 | 0.95 | 0.33 | 3.561 | 5.553 | 4.473 | 9.491 | 5.016 | 6.031 | -74.514 | 0.185 | 3.18 | 1.275 | 2.41 |
| Mean | -70.963 | 0.957 | 0.328 | 3.599 | 5.611 | 4.485 | 9.495 | 5.071 | 5.981 | -74.558 | 0.187 | 3.166 | 1.27 | 2.384 |
| BF | 7.368 |  |  |  |  |  |  |  |  |  |  |  |  |  |

Gelman-Rubin MCMC chain diagnostic test

|  | Point est. | Upper C.I. |
| --- | --- | --- |
| Dependent | 1 | 1 |
| Independent | 1 | 1 |

Table S30: Austronesian : Eskimo <-> Neo.localresidence

| run | MLL | q12 | q13 | q21 | q24 | q31 | q34 | q42 | q43 | MLL | α1 | β1 | α2 | β2 |
| --- | --- | --- | --- | --- | --- | --- | --- | --- | --- | --- | --- | --- | --- | --- |
| 1 | -52.993 | 0.19 | 0.207 | 8.934 | 10.115 | 3.249 | 4.742 | 6.91 | 11.153 | -56.985 | 0.166 | 3.223 | 0.619 | 7.221 |
| 2 | -52.876 | 0.188 | 0.205 | 8.96 | 10.336 | 3.262 | 4.683 | 6.987 | 10.939 | -56.776 | 0.169 | 3.241 | 0.618 | 7.133 |
| 3 | -52.852 | 0.189 | 0.202 | 8.97 | 10.232 | 3.233 | 4.714 | 6.961 | 11.16 | -56.722 | 0.165 | 3.214 | 0.619 | 7.184 |
| Mean | -52.907 | 0.189 | 0.205 | 8.955 | 10.228 | 3.248 | 4.713 | 6.953 | 11.084 | -56.828 | 0.167 | 3.226 | 0.619 | 7.179 |
| BF | 7.984 |  |  |  |  |  |  |  |  |  |  |  |  |  |

Gelman-Rubin MCMC chain diagnostic test

|  | Point est. | Upper C.I. |
| --- | --- | --- |
| Dependent | 1.000 | 1.001 |
| Independent | 1.001 | 1.002 |

Table S31: Austronesian : Eskimo <-> Nuclear.families

| run | MLL | q12 | q13 | q21 | q24 | q31 | q34 | q42 | q43 | MLL | α1 | β1 | α2 | β2 |
| --- | --- | --- | --- | --- | --- | --- | --- | --- | --- | --- | --- | --- | --- | --- |
| 1 | -88.88 | 4.445 | 0.542 | 6.428 | 1.792 | 7.766 | 10.399 | 4.003 | 3.262 | -89.35 | 0.17 | 3.154 | 3.983 | 4.046 |
| 2 | -89.04 | 4.413 | 0.539 | 6.373 | 1.827 | 7.626 | 10.282 | 4.013 | 3.18 | -89.26 | 0.167 | 3.167 | 3.965 | 4.042 |
| 3 | -88.89 | 4.394 | 0.531 | 6.483 | 1.735 | 7.539 | 10.413 | 4.001 | 3.202 | -89.11 | 0.17 | 3.169 | 3.954 | 4.029 |
| Mean | -88.93 | 4.417 | 0.537 | 6.428 | 1.785 | 7.644 | 10.365 | 4.006 | 3.215 | -89.24 | 0.169 | 3.163 | 3.967 | 4.039 |
| BF | 0.963 |  |  |  |  |  |  |  |  |  |  |  |  |  |

Gelman-Rubin MCMC chain diagnostic test

|  | Point est. | Upper C.I. |
| --- | --- | --- |
| Dependent | 1.001 | 1.002 |
| Independent | 1.001 | 1.002 |

Table S32: Austronesian : Eskimo <-> Uni.linealdescent

| run | MLL | q12 | q13 | q21 | q24 | q31 | q34 | q42 | q43 | MLL | α1 | β1 | α2 | β2 |
| --- | --- | --- | --- | --- | --- | --- | --- | --- | --- | --- | --- | --- | --- | --- |
| 1 | -86.068 | 2.3 | 0.518 | 3.44 | 0.558 | 3.108 | 1.524 | 11.284 | 10.553 | -85.802 | 0.179 | 3.218 | 1.437 | 3.442 |
| 2 | -86.834 | 2.335 | 0.507 | 3.427 | 0.553 | 3.098 | 1.531 | 11.294 | 10.388 | -85.815 | 0.188 | 3.228 | 1.443 | 3.433 |
| 3 | -86.23 | 2.326 | 0.515 | 3.428 | 0.562 | 3.142 | 1.5 | 11.261 | 10.464 | -85.894 | 0.178 | 3.249 | 1.489 | 3.432 |
| Mean | -86.377 | 2.32 | 0.513 | 3.432 | 0.558 | 3.116 | 1.518 | 11.28 | 10.468 | -85.837 | 0.182 | 3.232 | 1.456 | 3.436 |
| BF | -0.532 |  |  |  |  |  |  |  |  |  |  |  |  |  |

Gelman-Rubin MCMC chain diagnostic test

|  | Point est. | Upper C.I. |
| --- | --- | --- |
| Dependent | 1 | 1.001 |
| Independent | 1 | 1.000 |

Table S33: Austronesian : Hawaiian <-> Absenceofcousinmarriage.permitted

| run | MLL | q12 | q13 | q21 | q24 | q31 | q34 | q42 | q43 | MLL | α1 | β1 | α2 | β2 |
| --- | --- | --- | --- | --- | --- | --- | --- | --- | --- | --- | --- | --- | --- | --- |
| 1 | -102.542 | 4.757 | 4.315 | 2.359 | 8.871 | 4.497 | 15.427 | 7.638 | 4.567 | -102.648 | 6.738 | 6.067 | 14.573 | 5.101 |
| 2 | -102.493 | 4.618 | 4.2 | 2.318 | 8.747 | 4.58 | 15.457 | 7.478 | 4.706 | -102.624 | 6.728 | 6.057 | 14.533 | 5.056 |
| 3 | -102.484 | 4.928 | 4.295 | 2.318 | 8.716 | 4.681 | 15.472 | 7.413 | 4.679 | -102.653 | 6.714 | 6.118 | 14.56 | 5.082 |
| Mean | -102.506 | 4.768 | 4.27 | 2.332 | 8.778 | 4.586 | 15.452 | 7.51 | 4.651 | -102.642 | 6.727 | 6.081 | 14.555 | 5.08 |
| BF | 0.211 |  |  |  |  |  |  |  |  |  |  |  |  |  |

Gelman-Rubin MCMC chain diagnostic test

|  | Point est. | Upper C.I. |
| --- | --- | --- |
| Dependent | 1 | 1.001 |
| Independent | 1 | 1.000 |

Table S34: Austronesian : Hawaiian <-> Absenceofcousinmarriage.preference

| run | MLL | q12 | q13 | q21 | q24 | q31 | q34 | q42 | q43 | MLL | α1 | β1 | α2 | β2 |
| --- | --- | --- | --- | --- | --- | --- | --- | --- | --- | --- | --- | --- | --- | --- |
| 1 | -90.287 | 5.743 | 8.441 | 15.65 | 3.337 | 5.406 | 1.791 | 15.294 | 18.575 | -92.997 | 6.826 | 6.077 | 3.729 | 19.744 |
| 2 | -90.123 | 5.701 | 8.469 | 15.488 | 3.263 | 5.415 | 1.751 | 14.794 | 18.612 | -92.902 | 6.923 | 6.194 | 3.669 | 19.448 |
| 3 | -90.152 | 5.76 | 8.46 | 15.71 | 3.36 | 5.442 | 1.798 | 14.817 | 18.572 | -92.962 | 6.762 | 6.043 | 3.688 | 19.664 |
| Mean | -90.187 | 5.735 | 8.457 | 15.616 | 3.32 | 5.421 | 1.78 | 14.968 | 18.586 | -92.954 | 6.837 | 6.105 | 3.695 | 19.619 |
| BF | 5.421 |  |  |  |  |  |  |  |  |  |  |  |  |  |

Gelman-Rubin MCMC chain diagnostic test

|  | Point est. | Upper C.I. |
| --- | --- | --- |
| Dependent | 1 | 1 |
| Independent | 1 | 1 |

Table S35: Austronesian : Hawaiian <-> Bi.linealdescent

| run | MLL | q12 | q13 | q21 | q24 | q31 | q34 | q42 | q43 | MLL | α1 | β1 | α2 | β2 |
| --- | --- | --- | --- | --- | --- | --- | --- | --- | --- | --- | --- | --- | --- | --- |
| 1 | -111.998 | 4.097 | 10.549 | 13.825 | 8.256 | 16.902 | 3.161 | 2.369 | 3.754 | -117.348 | 7.679 | 6.872 | 2.454 | 4.274 |
| 2 | -111.94 | 4.077 | 10.469 | 13.909 | 8.299 | 16.719 | 3.226 | 2.428 | 3.772 | -117.297 | 7.696 | 6.824 | 2.461 | 4.253 |
| 3 | -111.932 | 4.107 | 10.525 | 13.756 | 8.355 | 16.86 | 3.097 | 2.39 | 3.732 | -117.44 | 7.782 | 6.855 | 2.451 | 4.272 |
| Mean | -111.957 | 4.094 | 10.514 | 13.83 | 8.303 | 16.827 | 3.161 | 2.396 | 3.753 | -117.362 | 7.719 | 6.85 | 2.455 | 4.266 |
| BF | 10.7 |  |  |  |  |  |  |  |  |  |  |  |  |  |

Gelman-Rubin MCMC chain diagnostic test

|  | Point est. | Upper C.I. |
| --- | --- | --- |
| Dependent | 1 | 1.000 |
| Independent | 1 | 1.001 |

Table S36: Austronesian : Hawaiian <-> Bi.localextendedfamily

| run | MLL | q12 | q13 | q21 | q24 | q31 | q34 | q42 | q43 | MLL | α1 | β1 | α2 | β2 |
| --- | --- | --- | --- | --- | --- | --- | --- | --- | --- | --- | --- | --- | --- | --- |
| 1 | -114.384 | 3.253 | 5.948 | 4.958 | 15.829 | 8.111 | 5.442 | 7.51 | 5.988 | -114.856 | 8.786 | 7.252 | 3.461 | 4.918 |
| 2 | -114.362 | 3.262 | 5.96 | 4.986 | 16.21 | 8.028 | 5.454 | 7.715 | 6.009 | -114.884 | 8.792 | 7.195 | 3.441 | 4.841 |
| 3 | -114.248 | 3.215 | 5.943 | 5.035 | 15.987 | 8.004 | 5.456 | 7.63 | 5.996 | -114.913 | 8.761 | 7.25 | 3.485 | 4.868 |
| Mean | -114.331 | 3.243 | 5.95 | 4.993 | 16.009 | 8.048 | 5.451 | 7.618 | 5.998 | -114.884 | 8.78 | 7.232 | 3.462 | 4.876 |
| BF | 0.945 |  |  |  |  |  |  |  |  |  |  |  |  |  |

Gelman-Rubin MCMC chain diagnostic test

|  | Point est. | Upper C.I. |
| --- | --- | --- |
| Dependent | 1.001 | 1.002 |
| Independent | 1.000 | 1.002 |

Table S37: Austronesian : Hawaiian <-> Bi.localresidence

| run | MLL | q12 | q13 | q21 | q24 | q31 | q34 | q42 | q43 | MLL | α1 | β1 | α2 | β2 |
| --- | --- | --- | --- | --- | --- | --- | --- | --- | --- | --- | --- | --- | --- | --- |
| 1 | -103.861 | 3.23 | 7.307 | 14.812 | 7.149 | 6.874 | 5.444 | 4.684 | 19.514 | -103.088 | 7.012 | 6.119 | 5.049 | 20.602 |
| 2 | -103.682 | 3.213 | 7.346 | 14.382 | 6.961 | 6.89 | 5.482 | 4.642 | 19.89 | -102.992 | 6.974 | 6.097 | 5.003 | 20.464 |
| 3 | -103.945 | 3.177 | 7.468 | 14.755 | 7.139 | 6.911 | 5.493 | 4.843 | 19.925 | -102.963 | 6.898 | 6.031 | 4.964 | 20.284 |
| Mean | -103.829 | 3.207 | 7.374 | 14.65 | 7.083 | 6.892 | 5.473 | 4.723 | 19.776 | -103.014 | 6.961 | 6.082 | 5.005 | 20.45 |
| BF | -1.547 |  |  |  |  |  |  |  |  |  |  |  |  |  |

Gelman-Rubin MCMC chain diagnostic test

|  | Point est. | Upper C.I. |
| --- | --- | --- |
| Dependent | 1 | 1.001 |
| Independent | 1 | 1.000 |

Table S38: Austronesian : Iroquois <-> Cross.cousinmarriage.permitted

| run | MLL | q12 | q13 | q21 | q24 | q31 | q34 | q42 | q43 | MLL | α1 | β1 | α2 | β2 |
| --- | --- | --- | --- | --- | --- | --- | --- | --- | --- | --- | --- | --- | --- | --- |
| 1 | -66.212 | 2.049 | 0.825 | 22.418 | 11.112 | 6.292 | 5.084 | 5.625 | 10.023 | -70.779 | 1.55 | 5.952 | 3.082 | 20.328 |
| 2 | -66.078 | 2.035 | 0.809 | 22.079 | 11.321 | 6.317 | 5.223 | 5.484 | 10.178 | -70.666 | 1.526 | 5.964 | 3.063 | 20.467 |
| 3 | -66.007 | 2.039 | 0.806 | 22.48 | 11.465 | 6.273 | 5.088 | 5.678 | 9.919 | -70.674 | 1.535 | 5.856 | 3.065 | 20.308 |
| Mean | -66.099 | 2.041 | 0.813 | 22.326 | 11.299 | 6.294 | 5.132 | 5.596 | 10.04 | -70.706 | 1.537 | 5.924 | 3.07 | 20.368 |
| BF | 9.135 |  |  |  |  |  |  |  |  |  |  |  |  |  |

Gelman-Rubin MCMC chain diagnostic test

|  | Point est. | Upper C.I. |
| --- | --- | --- |
| Dependent | 1 | 1 |
| Independent | 1 | 1 |

Table S39: Austronesian : Iroquois <-> Cross.cousinmarriage.preferred

| run | MLL | q12 | q13 | q21 | q24 | q31 | q34 | q42 | q43 | MLL | α1 | β1 | α2 | β2 |
| --- | --- | --- | --- | --- | --- | --- | --- | --- | --- | --- | --- | --- | --- | --- |
| 1 | -89.481 | 7.937 | 0.769 | 14.233 | 3.791 | 10.733 | 12.362 | 6.278 | 2.794 | -94.37 | 1.526 | 5.851 | 9.951 | 11.869 |
| 2 | -89.475 | 7.963 | 0.792 | 14.414 | 3.715 | 10.917 | 12.374 | 6.15 | 2.8 | -94.26 | 1.524 | 5.854 | 10.026 | 12.15 |
| 3 | -89.216 | 7.93 | 0.795 | 14.286 | 3.696 | 10.931 | 12.209 | 6.159 | 2.797 | -94.27 | 1.509 | 5.798 | 10.106 | 12.134 |
| Mean | -89.391 | 7.943 | 0.785 | 14.311 | 3.734 | 10.86 | 12.315 | 6.196 | 2.797 | -94.3 | 1.52 | 5.834 | 10.028 | 12.051 |
| BF | 9.785 |  |  |  |  |  |  |  |  |  |  |  |  |  |

Gelman-Rubin MCMC chain diagnostic test

|  | Point est. | Upper C.I. |
| --- | --- | --- |
| Dependent | 1 | 1 |
| Independent | 1 | 1 |

Table S40: Austronesian : Iroquois <-> Exogamy.unilineal.descent

| run | MLL | q12 | q13 | q21 | q24 | q31 | q34 | q42 | q43 | MLL | α1 | β1 | α2 | β2 |
| --- | --- | --- | --- | --- | --- | --- | --- | --- | --- | --- | --- | --- | --- | --- |
| 1 | -53.48 | 0.896 | 1.035 | 16.672 | 8.804 | 5.495 | 2.883 | 5.831 | 15.25 | -53.582 | 1.143 | 5.231 | 1.199 | 18.629 |
| 2 | -53.262 | 0.876 | 1.037 | 16.762 | 8.615 | 5.446 | 2.896 | 5.71 | 15.16 | -53.591 | 1.131 | 5.157 | 1.189 | 18.488 |
| 3 | -53.163 | 0.882 | 1.019 | 16.582 | 8.725 | 5.46 | 2.866 | 5.695 | 14.919 | -53.578 | 1.122 | 5.166 | 1.193 | 18.442 |
| Mean | -53.302 | 0.885 | 1.03 | 16.672 | 8.715 | 5.467 | 2.882 | 5.745 | 15.11 | -53.584 | 1.132 | 5.185 | 1.194 | 18.52 |
| BF | 0.205 |  |  |  |  |  |  |  |  |  |  |  |  |  |

Gelman-Rubin MCMC chain diagnostic test

|  | Point est. | Upper C.I. |
| --- | --- | --- |
| Dependent | 1 | 1 |
| Independent | 1 | 1 |

Table S41: Austronesian : Iroquois <-> High.polygyny

| run | MLL | q12 | q13 | q21 | q24 | q31 | q34 | q42 | q43 | MLL | α1 | β1 | α2 | β2 |
| --- | --- | --- | --- | --- | --- | --- | --- | --- | --- | --- | --- | --- | --- | --- |
| 1 | -67.953 | 2.031 | 1.149 | 15.69 | 6.505 | 7.702 | 5.548 | 8.159 | 10.349 | -69.217 | 1.711 | 7.769 | 2.754 | 15.809 |
| 2 | -67.932 | 2.021 | 1.172 | 15.766 | 6.534 | 7.687 | 5.453 | 8.273 | 10.587 | -69.048 | 1.701 | 7.852 | 2.806 | 16.062 |
| 3 | -67.949 | 2.061 | 1.179 | 16.003 | 6.545 | 7.741 | 5.423 | 8.483 | 10.216 | -69.301 | 1.698 | 7.807 | 2.753 | 15.811 |
| Mean | -67.945 | 2.038 | 1.167 | 15.82 | 6.528 | 7.71 | 5.475 | 8.305 | 10.384 | -69.189 | 1.703 | 7.809 | 2.771 | 15.894 |
| BF | 2.528 |  |  |  |  |  |  |  |  |  |  |  |  |  |

Gelman-Rubin MCMC chain diagnostic test

|  | Point est. | Upper C.I. |
| --- | --- | --- |
| Dependent | 1 | 1 |
| Independent | 1 | 1 |

Table S42: Austronesian : Iroquois <-> Matri.anvunclocalresidence

| run | MLL | q12 | q13 | q21 | q24 | q31 | q34 | q42 | q43 | MLL | α1 | β1 | α2 | β2 |
| --- | --- | --- | --- | --- | --- | --- | --- | --- | --- | --- | --- | --- | --- | --- |
| 1 | -73.87 | 1.383 | 0.844 | 7.123 | 3.16 | 4.147 | 1.605 | 11.378 | 8.102 | -72.25 | 1.045 | 4.985 | 1.153 | 5.678 |
| 2 | -73.80 | 1.4 | 0.841 | 7.184 | 3.167 | 4.198 | 1.606 | 11.468 | 8.018 | -72.407 | 1.055 | 5.016 | 1.166 | 5.737 |
| 3 | -73.70 | 1.39 | 0.856 | 7.093 | 3.163 | 4.189 | 1.608 | 11.213 | 8.029 | -72.18 | 1.049 | 4.976 | 1.16 | 5.625 |
| Mean | -73.79 | 1.391 | 0.847 | 7.133 | 3.163 | 4.178 | 1.606 | 11.353 | 8.05 | -72.279 | 1.05 | 4.992 | 1.16 | 5.68 |
| BF | -3.243 |  |  |  |  |  |  |  |  |  |  |  |  |  |

Gelman-Rubin MCMC chain diagnostic test

|  | Point est. | Upper C.I. |
| --- | --- | --- |
| Dependent | 1 | 1 |
| Independent | 1 | 1 |

Table S43: Austronesian : Iroquois <-> Polygyny

| run | MLL | q12 | q13 | q21 | q24 | q31 | q34 | q42 | q43 | MLL | α1 | β1 | α2 | β2 |
| --- | --- | --- | --- | --- | --- | --- | --- | --- | --- | --- | --- | --- | --- | --- |
| 1 | -78.608 | 1.915 | 1.063 | 1.325 | 2.521 | 11.089 | 11.082 | 9.884 | 3.903 | -77.895 | 1.695 | 7.788 | 2.586 | 1.562 |
| 2 | -78.508 | 1.919 | 1.061 | 1.331 | 2.566 | 10.846 | 11.184 | 10.011 | 3.916 | -77.924 | 1.708 | 7.843 | 2.628 | 1.561 |
| 3 | -78.393 | 1.914 | 1.078 | 1.328 | 2.6 | 10.918 | 11.112 | 10.047 | 3.865 | -77.762 | 1.69 | 7.83 | 2.611 | 1.571 |
| Mean | -78.503 | 1.916 | 1.067 | 1.328 | 2.562 | 10.951 | 11.126 | 9.981 | 3.895 | -77.86 | 1.698 | 7.82 | 2.608 | 1.565 |
| BF | -1.425 |  |  |  |  |  |  |  |  |  |  |  |  |  |

Gelman-Rubin MCMC chain diagnostic test

|  | Point est. | Upper C.I. |
| --- | --- | --- |
| Dependent | 1.001 | 1.001 |
| Independent | 1.000 | 1.000 |

Table S44: Austronesian : Iroquois <-> Uni.linealdescent

| run | MLL | q12 | q13 | q21 | q24 | q31 | q34 | q42 | q43 | MLL | α1 | β1 | α2 | β2 |
| --- | --- | --- | --- | --- | --- | --- | --- | --- | --- | --- | --- | --- | --- | --- |
| 1 | -87.18 | 0.937 | 0.834 | 4.408 | 5.463 | 6.662 | 10.617 | 9.374 | 4.777 | -90.726 | 1.502 | 6.014 | 1.055 | 3.397 |
| 2 | -87.37 | 0.934 | 0.827 | 4.447 | 5.421 | 6.419 | 10.811 | 9.27 | 4.666 | -90.821 | 1.492 | 5.929 | 1.095 | 3.389 |
| 3 | -87.08 | 0.936 | 0.829 | 4.423 | 5.524 | 6.446 | 10.838 | 9.442 | 4.75 | -90.937 | 1.518 | 6 | 1.07 | 3.398 |
| Mean | 87.21 | 0.936 | 0.83 | 4.426 | 5.469 | 6.509 | 10.755 | 9.362 | 4.731 | -90.828 | 1.504 | 5.981 | 1.073 | 3.395 |
| BF | 7.101 |  |  |  |  |  |  |  |  |  |  |  |  |  |

Gelman-Rubin MCMC chain diagnostic test

|  | Point est. | Upper C.I. |
| --- | --- | --- |
| Dependent | 1 | 1 |
| Independent | 1 | 1 |

Table S45: Austronesian : Iroquois <-> Uni.localresidence

| run | MLL | q12 | q13 | q21 | q24 | q31 | q34 | q42 | q43 | MLL | α1 | β1 | α2 | β2 |
| --- | --- | --- | --- | --- | --- | --- | --- | --- | --- | --- | --- | --- | --- | --- |
| 1 | -84.078 | 1.539 | 0.424 | 3.218 | 5.365 | 8.071 | 6.828 | 9.224 | 2.713 | -87.299 | 1.046 | 5.167 | 1.408 | 2.469 |
| 2 | -83.976 | 1.563 | 0.427 | 3.229 | 5.295 | 8.036 | 6.488 | 9.061 | 2.638 | -87.331 | 1.054 | 5.161 | 1.408 | 2.485 |
| 3 | -83.984 | 1.548 | 0.423 | 3.243 | 5.295 | 8.168 | 6.755 | 9.119 | 2.728 | -87.242 | 1.03 | 5.111 | 1.393 | 2.483 |
| Mean | -84.013 | 1.55 | 0.425 | 3.23 | 5.318 | 8.092 | 6.69 | 9.135 | 2.693 | -87.291 | 1.043 | 5.146 | 1.403 | 2.479 |
| BF | 6.441 |  |  |  |  |  |  |  |  |  |  |  |  |  |

Gelman-Rubin MCMC chain diagnostic test

|  | Point est. | Upper C.I. |
| --- | --- | --- |
| Dependent | 1 | 1.001 |
| Independent | 1 | 1.001 |

Table S46: Bantu : Crow <-> Matri.anvunclocalresidence

| run | MLL | q12 | q13 | q21 | q24 | q31 | q34 | q42 | q43 | MLL | α1 | β1 | α2 | β2 |
| --- | --- | --- | --- | --- | --- | --- | --- | --- | --- | --- | --- | --- | --- | --- |
| 1 | -63.233 | 3.683 | 0.933 | 8.457 | 2.134 | 16.453 | 7.622 | 14.886 | 7.657 | -62.465 | 1.269 | 17.921 | 4.049 | 8.668 |
| 2 | -63.222 | 3.759 | 0.931 | 8.619 | 2.118 | 15.965 | 7.62 | 14.76 | 7.5 | -62.369 | 1.267 | 17.875 | 4.082 | 8.768 |
| 3 | -63.23 | 3.744 | 0.936 | 8.622 | 2.106 | 16.082 | 7.723 | 14.656 | 7.519 | -62.411 | 1.308 | 18.338 | 4.028 | 8.593 |
| Mean | -63.228 | 3.729 | 0.933 | 8.566 | 2.119 | 16.167 | 7.655 | 14.767 | 7.559 | -62.415 | 1.281 | 18.045 | 4.053 | 8.676 |
| BF | -1.536 |  |  |  |  |  |  |  |  |  |  |  |  |  |

Gelman-Rubin MCMC chain diagnostic test

|  | Point est. | Upper C.I. |
| --- | --- | --- |
| Dependent | 1 | 1 |
| Independent | 1 | 1 |

Table S47: Bantu : Crow <-> Matrilineal

| run | MLL | q12 | q13 | q21 | q24 | q31 | q34 | q42 | q43 | MLL | α1 | β1 | α2 | β2 |
| --- | --- | --- | --- | --- | --- | --- | --- | --- | --- | --- | --- | --- | --- | --- |
| 1 | -63.159 | 2.092 | 1.131 | 3.989 | 1.792 | 16.983 | 7.885 | 15.932 | 7.459 | -62.249 | 1.276 | 18.031 | 2.289 | 4.109 |
| 2 | -63.343 | 2.107 | 1.11 | 3.97 | 1.804 | 17.001 | 7.864 | 15.723 | 7.361 | -62.244 | 1.293 | 18.128 | 2.326 | 4.177 |
| 3 | -63.289 | 2.124 | 1.132 | 3.955 | 1.791 | 17.093 | 7.848 | 15.781 | 7.493 | -62.241 | 1.272 | 17.898 | 2.276 | 4.119 |
| Mean | -63.264 | 2.108 | 1.124 | 3.971 | 1.796 | 17.026 | 7.866 | 15.812 | 7.438 | -62.245 | 1.28 | 18.019 | 2.297 | 4.135 |
| BF | -1.82 |  |  |  |  |  |  |  |  |  |  |  |  |  |

Gelman-Rubin MCMC chain diagnostic test

|  | Point est. | Upper C.I. |
| --- | --- | --- |
| Dependent | 1 | 1.001 |
| Independent | 1 | 1.000 |

Table S48: Bantu : Crow <-> Matrilocal

| run | MLL | q12 | q13 | q21 | q24 | q31 | q34 | q42 | q43 | MLL | α1 | β1 | α2 | β2 |
| --- | --- | --- | --- | --- | --- | --- | --- | --- | --- | --- | --- | --- | --- | --- |
| 1 | -36.08 | 0.844 | 0.861 | 15.206 | 5.526 | 15.506 | 5.245 | 11.085 | 11.464 | -36.34 | 1.275 | 17.931 | 1.203 | 16.955 |
| 2 | -35.87 | 0.849 | 0.903 | 15.359 | 5.536 | 16.053 | 5.295 | 11.284 | 11.188 | -36.25 | 1.268 | 17.936 | 1.197 | 16.809 |
| 3 | -35.96 | 0.868 | 0.878 | 15.47 | 5.526 | 15.841 | 5.241 | 11.172 | 11.245 | -36.28 | 1.254 | 17.755 | 1.195 | 16.949 |
| Mean | -35.97 | 0.854 | 0.881 | 15.345 | 5.529 | 15.8 | 5.26 | 11.18 | 11.299 | -36.29 | 1.266 | 17.874 | 1.198 | 16.904 |
| BF | 0.523 |  |  |  |  |  |  |  |  |  |  |  |  |  |

Gelman-Rubin MCMC chain diagnostic test

|  | Point est. | Upper C.I. |
| --- | --- | --- |
| Dependent | 1 | 1 |
| Independent | 1 | 1 |

Table S49: Bantu : Crow <-> Matrilocal.strict

| run | MLL | q12 | q13 | q21 | q24 | q31 | q34 | q42 | q43 | MLL | α1 | β1 | α2 | β2 |
| --- | --- | --- | --- | --- | --- | --- | --- | --- | --- | --- | --- | --- | --- | --- |
| 1 | -36.04 | 0.86 | 0.861 | 15.297 | 5.524 | 15.811 | 5.204 | 11.074 | 11.242 | -36.42 | 1.258 | 17.746 | 1.198 | 16.758 |
| 2 | -35.91 | 0.861 | 0.862 | 15.371 | 5.578 | 15.86 | 5.285 | 11.54 | 11.216 | -36.36 | 1.271 | 17.976 | 1.18 | 16.774 |
| 3 | -36.16 | 0.897 | 0.875 | 15.562 | 5.606 | 15.799 | 5.05 | 11.536 | 11.234 | -36.31 | 1.255 | 18.041 | 1.191 | 16.829 |
| Mean | -36.03 | 0.873 | 0.866 | 15.41 | 5.569 | 15.823 | 5.18 | 11.383 | 11.231 | -36.36 | 1.261 | 17.921 | 1.19 | 16.787 |
| BF | 0.753 |  |  |  |  |  |  |  |  |  |  |  |  |  |

Gelman-Rubin MCMC chain diagnostic test

|  | Point est. | Upper C.I. |
| --- | --- | --- |
| Dependent | 1 | 1.001 |
| Independent | 1 | 1.000 |

Table S50: Bantu : Crow <-> Uni.localresidence

| run | MLL | q12 | q13 | q21 | q24 | q31 | q34 | q42 | q43 | MLL | α1 | β1 | α2 | β2 |
| --- | --- | --- | --- | --- | --- | --- | --- | --- | --- | --- | --- | --- | --- | --- |
| 1 | -56.96 | 19.022 | 1.881 | 4.725 | 1.07 | 13.671 | 10.166 | 16.189 | 5.362 | -55.84 | 1.283 | 18.04 | 18.558 | 4.924 |
| 2 | -57.01 | 18.995 | 1.907 | 4.755 | 1.065 | 13.61 | 10.374 | 16.133 | 5.571 | -55.88 | 1.26 | 17.698 | 18.951 | 4.893 |
| 3 | -56.89 | 19.072 | 1.916 | 4.736 | 1.058 | 13.501 | 10.363 | 16.241 | 5.434 | -55.97 | 1.275 | 18.058 | 18.664 | 4.879 |
| Mean | -56.95 | 19.03 | 1.901 | 4.739 | 1.064 | 13.594 | 10.301 | 16.188 | 5.456 | -55.9 | 1.273 | 17.932 | 18.724 | 4.899 |
| BF | -2.231 |  |  |  |  |  |  |  |  |  |  |  |  |  |

Gelman-Rubin MCMC chain diagnostic test

|  | Point est. | Upper C.I. |
| --- | --- | --- |
| Dependent | 1 | 1 |
| Independent | 1 | 1 |

Table S51: Bantu : Hawaiian <-> Absenceofcousinmarriage.permitted

| run | MLL | q12 | q13 | q21 | q24 | q31 | q34 | q42 | q43 | MLL | α1 | β1 | α2 | β2 |
| --- | --- | --- | --- | --- | --- | --- | --- | --- | --- | --- | --- | --- | --- | --- |
| 1 | -58.393 | 14.028 | 3.116 | 2.389 | 1.2 | 6.522 | 9.485 | 5.252 | 4.025 | -57.357 | 1.021 | 2.536 | 12.158 | 2.326 |
| 2 | -58.324 | 14.076 | 3.103 | 2.382 | 1.212 | 6.787 | 9.544 | 5.329 | 4.035 | -57.332 | 1.007 | 2.43 | 12.03 | 2.333 |
| 3 | -58.377 | 13.979 | 3.153 | 2.362 | 1.191 | 6.621 | 9.334 | 5.212 | 4.096 | -57.241 | 1.018 | 2.582 | 11.983 | 2.364 |
| Mean | -58.365 | 14.028 | 3.124 | 2.378 | 1.201 | 6.643 | 9.454 | 5.264 | 4.052 | -57.31 | 1.015 | 2.516 | 12.057 | 2.341 |
| BF | -2.07 |  |  |  |  |  |  |  |  |  |  |  |  |  |

Gelman-Rubin MCMC chain diagnostic test

|  | Point est. | Upper C.I. |
| --- | --- | --- |
| Dependent | 1 | 1.000 |
| Independent | 1 | 1.001 |

Table S52: Bantu : Hawaiian <-> Absenceofcousinmarriage.preference

| run | MLL | q12 | q13 | q21 | q24 | q31 | q34 | q42 | q43 | MLL | α1 | β1 | α2 | β2 |
| --- | --- | --- | --- | --- | --- | --- | --- | --- | --- | --- | --- | --- | --- | --- |
| 1 | -56.612 | 2.768 | 1.112 | 14.599 | 1.733 | 2.826 | 1.422 | 10.263 | 14.536 | -55.227 | 1.006 | 2.518 | 2.53 | 15.173 |
| 2 | -56.706 | 2.791 | 1.087 | 14.687 | 1.776 | 2.711 | 1.445 | 10.097 | 14.638 | -55.367 | 1.015 | 2.448 | 2.519 | 15.194 |
| 3 | -56.672 | 2.809 | 1.094 | 14.769 | 1.773 | 2.802 | 1.439 | 10.178 | 14.584 | -55.352 | 1.007 | 2.43 | 2.511 | 15.11 |
| Mean | -56.663 | 2.789 | 1.098 | 14.685 | 1.761 | 2.78 | 1.435 | 10.179 | 14.586 | -55.315 | 1.009 | 2.465 | 2.52 | 15.159 |
| BF | -2.77 |  |  |  |  |  |  |  |  |  |  |  |  |  |

Gelman-Rubin MCMC chain diagnostic test

|  | Point est. | Upper C.I. |
| --- | --- | --- |
| Dependent | 1 | 1.000 |
| Independent | 1 | 1.001 |

Table S53: Bantu : Hawaiian <-> Bi.linealdescent

| run | MLL | q12 | q13 | q21 | q24 | q31 | q34 | q42 | q43 | MLL | α1 | β1 | α2 | β2 |
| --- | --- | --- | --- | --- | --- | --- | --- | --- | --- | --- | --- | --- | --- | --- |
| 1 | -60.055 | 3.092 | 1.279 | 18.826 | 3.927 | 7.162 | 4.153 | 8.473 | 11.61 | -59.335 | 1.391 | 6.346 | 3.362 | 18.639 |
| 2 | -60.145 | 3.111 | 1.267 | 18.837 | 3.904 | 7.084 | 4.184 | 8.717 | 11.652 | -59.389 | 1.377 | 6.284 | 3.402 | 18.483 |
| 3 | -59.917 | 3.097 | 1.263 | 18.648 | 3.827 | 7.109 | 4.065 | 8.385 | 11.312 | -59.293 | 1.376 | 6.261 | 3.316 | 18.364 |
| Mean | -60.039 | 3.1 | 1.27 | 18.77 | 3.886 | 7.118 | 4.134 | 8.525 | 11.525 | -59.339 | 1.381 | 6.297 | 3.36 | 18.495 |
| BF | -1.44 |  |  |  |  |  |  |  |  |  |  |  |  |  |

Gelman-Rubin MCMC chain diagnostic test

|  | Point est. | Upper C.I. |
| --- | --- | --- |
| Dependent | 1 | 1.000 |
| Independent | 1 | 1.001 |

Table S54: Bantu : Hawaiian <-> Bi.localextendedfamily

| run | MLL | q12 | q13 | q21 | q24 | q31 | q34 | q42 | q43 | MLL | α1 | β1 | α2 | β2 |
| --- | --- | --- | --- | --- | --- | --- | --- | --- | --- | --- | --- | --- | --- | --- |
| 1 | -71.5 | 6.828 | 1.406 | 20.283 | 2.408 | 7.765 | 5.858 | 7.939 | 9.5 | -70.622 | 1.388 | 6.392 | 7.039 | 19.43 |
| 2 | -71.426 | 6.783 | 1.456 | 20.199 | 2.395 | 8.138 | 5.929 | 7.728 | 9.358 | -70.605 | 1.365 | 6.244 | 7.034 | 19.285 |
| 3 | -71.384 | 6.853 | 1.426 | 20.222 | 2.376 | 7.822 | 5.895 | 7.779 | 9.483 | -70.642 | 1.369 | 6.021 | 7.05 | 19.347 |
| Mean | -71.437 | 6.821 | 1.429 | 20.235 | 2.393 | 7.908 | 5.894 | 7.815 | 9.447 | -70.623 | 1.374 | 6.219 | 7.041 | 19.354 |
| BF | -1.756 |  |  |  |  |  |  |  |  |  |  |  |  |  |

Gelman-Rubin MCMC chain diagnostic test

|  | Point est. | Upper C.I. |
| --- | --- | --- |
| Dependent | 1 | 1.000 |
| Independent | 1 | 1.001 |

Table S55: Bantu : Hawaiian <-> Bi.localresidence

| run | MLL | q12 | q13 | q21 | q24 | q31 | q34 | q42 | q43 | MLL | α1 | β1 | α2 | β2 |
| --- | --- | --- | --- | --- | --- | --- | --- | --- | --- | --- | --- | --- | --- | --- |
| 1 | -68.9 | 2.27 | 1.632 | 4.953 | 1.811 | 7.149 | 2.989 | 11.692 | 12.583 | -67.852 | 1.386 | 6.373 | 2.181 | 5.407 |
| 2 | -69.01 | 2.262 | 1.661 | 4.806 | 1.82 | 7.435 | 2.975 | 11.673 | 12.592 | -67.97 | 1.397 | 6.322 | 2.182 | 5.301 |
| 3 | -68.922 | 2.273 | 1.681 | 4.824 | 1.809 | 7.429 | 2.936 | 11.755 | 12.461 | -67.922 | 1.373 | 6.139 | 2.194 | 5.452 |
| Mean | -68.944 | 2.268 | 1.658 | 4.861 | 1.813 | 7.338 | 2.967 | 11.707 | 12.545 | -67.915 | 1.385 | 6.278 | 2.186 | 5.387 |
| BF | -2.097 |  |  |  |  |  |  |  |  |  |  |  |  |  |

Gelman-Rubin MCMC chain diagnostic test

|  | Point est. | Upper C.I. |
| --- | --- | --- |
| Dependent | 1 | 1.000 |
| Independent | 1 | 1.001 |

Table S56: Bantu : Iroquois <-> Cross.cousinmarriage.permitted

| run | MLL | q12 | q13 | q21 | q24 | q31 | q34 | q42 | q43 | MLL | α1 | β1 | α2 | β2 |
| --- | --- | --- | --- | --- | --- | --- | --- | --- | --- | --- | --- | --- | --- | --- |
| 1 | -73.417 | 1.107 | 2.71 | 14.119 | 10.485 | 3.136 | 3.914 | 2.567 | 14.346 | -73.483 | 2.288 | 2.572 | 2.714 | 14.219 |
| 2 | -73.314 | 1.095 | 2.729 | 13.652 | 10.269 | 3.206 | 3.845 | 2.518 | 14.117 | -73.595 | 2.314 | 2.593 | 2.696 | 14.281 |
| 3 | -73.237 | 1.113 | 2.689 | 13.827 | 10.36 | 3.179 | 3.812 | 2.48 | 14.081 | -73.513 | 2.375 | 2.604 | 2.704 | 14.424 |
| Mean | -73.323 | 1.105 | 2.709 | 13.866 | 10.371 | 3.174 | 3.857 | 2.522 | 14.181 | -73.53 | 2.326 | 2.59 | 2.705 | 14.308 |
| BF | 0.132 |  |  |  |  |  |  |  |  |  |  |  |  |  |

Gelman-Rubin MCMC chain diagnostic test

|  | Point est. | Upper C.I. |
| --- | --- | --- |
| Dependent | 1.001 | 1.001 |
| Independent | 1.000 | 1.000 |

Table S57: Bantu : Iroquois <-> Cross.cousinmarriage.preferred

| run | MLL | q12 | q13 | q21 | q24 | q31 | q34 | q42 | q43 | MLL | α1 | β1 | α2 | β2 |
| --- | --- | --- | --- | --- | --- | --- | --- | --- | --- | --- | --- | --- | --- | --- |
| 1 | -80.57 | 3.105 | 1.476 | 10.029 | 8.312 | 6.022 | 9.368 | 2.798 | 2.642 | -87.31 | 2.275 | 2.585 | 5.947 | 3.964 |
| 2 | -80.49 | 3.045 | 1.484 | 9.947 | 8.365 | 6.102 | 9.606 | 2.81 | 2.659 | -87.39 | 2.308 | 2.597 | 6.08 | 3.981 |
| 3 | -80.37 | 3.151 | 1.528 | 9.959 | 8.302 | 6.21 | 9.332 | 2.794 | 2.684 | -87.29 | 2.335 | 2.59 | 6.12 | 4.002 |
| Mean | -80.48 | 3.1 | 1.496 | 9.978 | 8.326 | 6.111 | 9.435 | 2.801 | 2.662 | -87.33 | 2.306 | 2.591 | 6.049 | 3.982 |
| BF | 13.494 |  |  |  |  |  |  |  |  |  |  |  |  |  |

Gelman-Rubin MCMC chain diagnostic test

|  | Point est. | Upper C.I. |
| --- | --- | --- |
| Dependent | 1 | 1 |
| Independent | 1 | 1 |

Table S58: Bantu : Iroquois <-> Exogamy.unilineal.descent

| run | MLL | q12 | q13 | q21 | q24 | q31 | q34 | q42 | q43 | MLL | α1 | β1 | α2 | β2 |
| --- | --- | --- | --- | --- | --- | --- | --- | --- | --- | --- | --- | --- | --- | --- |
| 1 | -70.066 | 3.594 | 2.12 | 16.39 | 7.682 | 2.787 | 2.428 | 4.407 | 13.797 | -69.06 | 2.257 | 2.65 | 3.112 | 16.955 |
| 2 | -70.024 | 3.645 | 2.124 | 16.476 | 7.545 | 2.803 | 2.456 | 4.317 | 13.538 | -69.042 | 2.223 | 2.598 | 3.108 | 16.877 |
| 3 | -70.061 | 3.636 | 2.106 | 16.683 | 7.722 | 2.771 | 2.395 | 4.41 | 13.785 | -69.048 | 2.239 | 2.61 | 3.118 | 16.955 |
| Mean | -70.05 | 3.625 | 2.117 | 16.516 | 7.65 | 2.787 | 2.426 | 4.378 | 13.707 | -69.05 | 2.24 | 2.619 | 3.113 | 16.929 |
| BF | -2.012 |  |  |  |  |  |  |  |  |  |  |  |  |  |

Gelman-Rubin MCMC chain diagnostic test

|  | Point est. | Upper C.I. |
| --- | --- | --- |
| Dependent | 1 | 1 |
| Independent | 1 | 1 |

Table S59: Bantu : Iroquois <-> Matri.anvunclocalresidence

| run | MLL | q12 | q13 | q21 | q24 | q31 | q34 | q42 | q43 | MLL | α1 | β1 | α2 | β2 |
| --- | --- | --- | --- | --- | --- | --- | --- | --- | --- | --- | --- | --- | --- | --- |
| 1 | -87.245 | 1.606 | 2.689 | 11.907 | 11.656 | 3.573 | 6.588 | 2.854 | 8.986 | -89.474 | 2.703 | 2.685 | 4.125 | 8.698 |
| 2 | -87.1 | 1.623 | 2.646 | 12.153 | 11.579 | 3.542 | 6.479 | 2.877 | 8.966 | -89.409 | 2.72 | 2.672 | 4.159 | 8.996 |
| 3 | -87.178 | 1.621 | 2.619 | 11.977 | 11.772 | 3.553 | 6.522 | 2.868 | 8.984 | -89.459 | 2.654 | 2.667 | 4.134 | 8.957 |
| Mean | -87.174 | 1.617 | 2.651 | 12.012 | 11.669 | 3.556 | 6.53 | 2.866 | 8.979 | -89.447 | 2.692 | 2.675 | 4.139 | 8.884 |
| BF | 4.458 |  |  |  |  |  |  |  |  |  |  |  |  |  |

Gelman-Rubin MCMC chain diagnostic test

|  | Point est. | Upper C.I. |
| --- | --- | --- |
| Dependent | 1 | 1 |
| Independent | 1 | 1 |

Table S60: Bantu : Iroquois <-> Uni.linealdescent

| run | MLL | q12 | q13 | q21 | q24 | q31 | q34 | q42 | q43 | MLL | α1 | β1 | α2 | β2 |
| --- | --- | --- | --- | --- | --- | --- | --- | --- | --- | --- | --- | --- | --- | --- |
| 1 | -79.123 | 14.122 | 5.772 | 3.675 | 3.359 | 6.596 | 17.602 | 2.675 | 3.188 | -78.33 | 2.712 | 2.702 | 17.789 | 3.581 |
| 2 | -79.051 | 14.266 | 5.791 | 3.697 | 3.441 | 6.584 | 17.413 | 2.652 | 3.246 | -78.218 | 2.685 | 2.678 | 17.753 | 3.561 |
| 3 | -79.209 | 14.273 | 5.842 | 3.684 | 3.468 | 6.728 | 17.444 | 2.662 | 3.203 | -78.202 | 2.676 | 2.669 | 17.646 | 3.539 |
| Mean | -79.128 | 14.22 | 5.802 | 3.685 | 3.423 | 6.636 | 17.486 | 2.663 | 3.212 | -78.25 | 2.691 | 2.683 | 17.729 | 3.56 |
| BF | -1.586 |  |  |  |  |  |  |  |  |  |  |  |  |  |

Gelman-Rubin MCMC chain diagnostic test

|  | Point est. | Upper C.I. |
| --- | --- | --- |
| Dependent | 1 | 1.000 |
| Independent | 1 | 1.001 |

Table S61: Bantu : Iroquois <-> Uni.localresidence

| run | MLL | q12 | q13 | q21 | q24 | q31 | q34 | q42 | q43 | MLL | α1 | β1 | α2 | β2 |
| --- | --- | --- | --- | --- | --- | --- | --- | --- | --- | --- | --- | --- | --- | --- |
| 1 | -84.198 | 14.459 | 7.409 | 3.398 | 3.372 | 3.4 | 18.168 | 3.191 | 5.269 | -82.955 | 2.689 | 2.672 | 18.782 | 4.922 |
| 2 | -84.14 | 14.678 | 7.317 | 3.396 | 3.275 | 3.449 | 18.311 | 3.193 | 5.265 | -82.853 | 2.67 | 2.674 | 18.834 | 5.005 |
| 3 | -83.9 | 14.514 | 7.441 | 3.334 | 3.324 | 3.462 | 18.109 | 3.176 | 5.303 | -82.932 | 2.635 | 2.638 | 18.933 | 4.963 |
| Mean | -84.079 | 14.55 | 7.389 | 3.376 | 3.324 | 3.437 | 18.196 | 3.187 | 5.279 | -82.913 | 2.665 | 2.661 | 18.85 | 4.963 |
| BF | -2.486 |  |  |  |  |  |  |  |  |  |  |  |  |  |

Gelman-Rubin MCMC chain diagnostic test

|  | Point est. | Upper C.I. |
| --- | --- | --- |
| Dependent | 1 | 1.000 |
| Independent | 1 | 1.001 |

Table S62: Bantu : Omaha <-> Matri.anvunclocalresidence

| run | MLL | q12 | q13 | q21 | q24 | q31 | q34 | q42 | q43 | MLL | α1 | β1 | α2 | β2 |
| --- | --- | --- | --- | --- | --- | --- | --- | --- | --- | --- | --- | --- | --- | --- |
| 1 | -70.562 | 4.869 | 2.25 | 8.616 | 0.739 | 10.969 | 1.765 | 14.163 | 12.403 | -70.678 | 1.627 | 11.749 | 4.135 | 8.9 |
| 2 | -70.529 | 4.81 | 2.229 | 8.691 | 0.728 | 10.911 | 1.788 | 13.862 | 12.767 | -70.725 | 1.633 | 11.804 | 4.079 | 8.662 |
| 3 | -70.488 | 4.819 | 2.23 | 8.647 | 0.737 | 10.861 | 1.84 | 14.007 | 12.381 | -70.834 | 1.631 | 11.71 | 4.096 | 8.824 |
| Mean | -70.526 | 4.833 | 2.236 | 8.651 | 0.735 | 10.914 | 1.798 | 14.011 | 12.517 | -70.746 | 1.63 | 11.754 | 4.103 | 8.795 |
| BF | 0.232 |  |  |  |  |  |  |  |  |  |  |  |  |  |

Gelman-Rubin MCMC chain diagnostic test

|  | Point est. | Upper C.I. |
| --- | --- | --- |
| Dependent | 1 | 1 |
| Independent | 1 | 1 |

Table S63: Bantu : Omaha <-> Patrilineal

| run | MLL | q12 | q13 | q21 | q24 | q31 | q34 | q42 | q43 | MLL | α1 | β1 | α2 | β2 |
| --- | --- | --- | --- | --- | --- | --- | --- | --- | --- | --- | --- | --- | --- | --- |
| 1 | -72.936 | 3.622 | 0.717 | 5.773 | 3.123 | 14.209 | 10.119 | 10.375 | 2.417 | -74.604 | 1.657 | 11.807 | 3.241 | 4.127 |
| 2 | -72.797 | 3.602 | 0.727 | 5.734 | 3.163 | 13.75 | 10.183 | 10.545 | 2.411 | -74.537 | 1.646 | 11.773 | 3.189 | 4.087 |
| 3 | -72.935 | 3.602 | 0.72 | 5.709 | 3.126 | 13.724 | 10.447 | 10.376 | 2.363 | -74.607 | 1.611 | 11.609 | 3.195 | 4.097 |
| Mean | -72.889 | 3.609 | 0.721 | 5.739 | 3.137 | 13.894 | 10.25 | 10.432 | 2.397 | -74.583 | 1.638 | 11.73 | 3.208 | 4.104 |
| BF | 3.336 |  |  |  |  |  |  |  |  |  |  |  |  |  |

Gelman-Rubin MCMC chain diagnostic test

|  | Point est. | Upper C.I. |
| --- | --- | --- |
| Dependent | 1 | 1.001 |
| Independent | 1 | 1.000 |

Table S64: Bantu : Omaha <-> Patrilocal

| run | MLL | q12 | q13 | q21 | q24 | q31 | q34 | q42 | q43 | MLL | α1 | β1 | α2 | β2 |
| --- | --- | --- | --- | --- | --- | --- | --- | --- | --- | --- | --- | --- | --- | --- |
| 1 | -69.50 | 19.426 | 0.871 | 8.128 | 1.91 | 13.444 | 13.605 | 10.198 | 1.899 | -69.16 | 1.632 | 11.707 | 19.964 | 7.246 |
| 2 | -69.45 | 19.311 | 0.915 | 8.118 | 1.952 | 13.63 | 13.447 | 10.14 | 1.902 | -69.13 | 1.631 | 11.667 | 19.949 | 7.178 |
| 3 | -69.39 | 19.431 | 0.884 | 8.182 | 1.954 | 13.6 | 13.468 | 10.218 | 1.925 | -69.10 | 1.625 | 11.573 | 20.107 | 7.234 |
| Mean | -69.44 | 19.389 | 0.89 | 8.143 | 1.939 | 13.558 | 13.507 | 10.185 | 1.909 | -69.13 | 1.629 | 11.649 | 20.007 | 7.219 |
| BF | -0.655 |  |  |  |  |  |  |  |  |  |  |  |  |  |

Gelman-Rubin MCMC chain diagnostic test

|  | Point est. | Upper C.I. |
| --- | --- | --- |
| Dependent | 1 | 1.001 |
| Independent | 1 | 1.001 |

Table S65: Bantu : Omaha <-> Patrilocal.strict

| run | MLL | q12 | q13 | q21 | q24 | q31 | q34 | q42 | q43 | MLL | α1 | β1 | α2 | β2 |
| --- | --- | --- | --- | --- | --- | --- | --- | --- | --- | --- | --- | --- | --- | --- |
| 1 | -70.708 | 2.91 | 0.426 | 4.629 | 3.429 | 14.016 | 11.818 | 11.12 | 1.737 | -73.92 | 1.628 | 11.779 | 2.79 | 3.553 |
| 2 | -70.764 | 2.906 | 0.426 | 4.645 | 3.423 | 13.832 | 11.776 | 11.117 | 1.64 | -73.886 | 1.617 | 11.611 | 2.775 | 3.556 |
| 3 | -70.881 | 2.896 | 0.424 | 4.63 | 3.458 | 14.38 | 11.936 | 11.011 | 1.721 | -73.766 | 1.613 | 11.584 | 2.777 | 3.573 |
| Mean | -70.784 | 2.904 | 0.425 | 4.635 | 3.437 | 14.076 | 11.843 | 11.083 | 1.699 | -73.857 | 1.619 | 11.658 | 2.781 | 3.561 |
| BF | 6.424 |  |  |  |  |  |  |  |  |  |  |  |  |  |

Gelman-Rubin MCMC chain diagnostic test

|  | Point est. | Upper C.I. |
| --- | --- | --- |
| Dependent | 1.001 | 1.003 |
| Independent | 1.001 | 1.002 |

Table S66: Bantu : Omaha <-> Uni.localresidence

| run | MLL | q12 | q13 | q21 | q24 | q31 | q34 | q42 | q43 | MLL | α1 | β1 | α2 | β2 |
| --- | --- | --- | --- | --- | --- | --- | --- | --- | --- | --- | --- | --- | --- | --- |
| 1 | -65.26 | 18.788 | 1.103 | 5.61 | 1.818 | 13.393 | 13.456 | 10.613 | 1.868 | -64.22 | 1.645 | 11.737 | 18.731 | 4.939 |
| 2 | -64.86 | 18.814 | 1.087 | 5.616 | 1.849 | 13.315 | 13.24 | 10.723 | 1.82 | -64.12 | 1.631 | 11.785 | 18.709 | 4.901 |
| 3 | -64.95 | 19.088 | 1.088 | 5.663 | 1.844 | 13.639 | 13.155 | 10.658 | 1.863 | -64.21 | 1.612 | 11.534 | 18.569 | 4.92 |
| Mean | -65.02 | 18.897 | 1.093 | 5.63 | 1.837 | 13.449 | 13.284 | 10.665 | 1.85 | -64.19 | 1.629 | 11.685 | 18.67 | 4.92 |
| BF | -2.09 |  |  |  |  |  |  |  |  |  |  |  |  |  |

Gelman-Rubin MCMC chain diagnostic test

|  | Point est. | Upper C.I. |
| --- | --- | --- |
| Dependent | 1 | 1.001 |
| Independent | 1 | 1.001 |

Table S67: Uto : Hawaiian <-> Absenceofcousinmarriage.permitted

| run | MLL | q12 | q13 | q21 | q24 | q31 | q34 | q42 | q43 | MLL | α1 | β1 | α2 | β2 |
| --- | --- | --- | --- | --- | --- | --- | --- | --- | --- | --- | --- | --- | --- | --- |
| 1 | -21.01 | 11.078 | 10.815 | 2.362 | 16.985 | 4.446 | 9.342 | 7.851 | 2.193 | -20.45 | 17.299 | 7.256 | 11.377 | 2.118 |
| 2 | -20.963 | 11.011 | 10.926 | 2.342 | 17.017 | 4.439 | 9.616 | 7.858 | 2.264 | -20.38 | 17.245 | 7.185 | 10.951 | 2.073 |
| 3 | -20.862 | 11.16 | 11.105 | 2.292 | 16.978 | 4.311 | 9.298 | 7.933 | 2.205 | -20.45 | 17.128 | 7.254 | 11.176 | 2.082 |
| Mean | -20.945 | 11.083 | 10.949 | 2.332 | 16.993 | 4.399 | 9.419 | 7.881 | 2.221 | -20.43 | 17.224 | 7.232 | 11.168 | 2.091 |
| BF | -1.114 |  |  |  |  |  |  |  |  |  |  |  |  |  |

Gelman-Rubin MCMC chain diagnostic test

|  | Point est. | Upper C.I. |
| --- | --- | --- |
| Dependent | 1.001 | 1.002 |
| Independent | 1.001 | 1.002 |

Table S68: Uto : Hawaiian <-> Absenceofcousinmarriage.preference

| run | MLL | q12 | q13 | q21 | q24 | q31 | q34 | q42 | q43 | MLL | α1 | β1 | α2 | β2 |
| --- | --- | --- | --- | --- | --- | --- | --- | --- | --- | --- | --- | --- | --- | --- |
| 1 | -20.186 | 2.033 | 17.534 | 11.31 | 9.369 | 7.466 | 1.974 | 5.066 | 17.712 | -19.37 | 17.279 | 7.291 | 1.785 | 18.447 |
| 2 | -20.141 | 2.068 | 17.475 | 11.169 | 9.319 | 7.557 | 1.95 | 4.952 | 17.707 | -19.33 | 17.13 | 7.251 | 1.757 | 18.322 |
| 3 | -20.17 | 2.014 | 17.439 | 10.877 | 9.037 | 7.476 | 1.919 | 4.849 | 17.433 | -19.29 | 17.197 | 7.234 | 1.804 | 18.584 |
| Mean | -20.166 | 2.038 | 17.483 | 11.119 | 9.242 | 7.5 | 1.948 | 4.956 | 17.617 | -19.33 | 17.202 | 7.259 | 1.782 | 18.451 |
| BF | -1.632 |  |  |  |  |  |  |  |  |  |  |  |  |  |

Gelman-Rubin MCMC chain diagnostic test

|  | Point est. | Upper C.I. |
| --- | --- | --- |
| Dependent | 1 | 1 |
| Independent | 1 | 1 |

Table S69: Uto : Hawaiian <-> Bi.linealdescent

| run | MLL | q12 | q13 | q21 | q24 | q31 | q34 | q42 | q43 | MLL | α1 | β1 | α2 | β2 |
| --- | --- | --- | --- | --- | --- | --- | --- | --- | --- | --- | --- | --- | --- | --- |
| 1 | -22.543 | 2.046 | 9.268 | 14.8 | 9.154 | 4.942 | 1.262 | 7.598 | 12.369 | -21.76 | 11.513 | 6.152 | 1.298 | 16.03 |
| 2 | -22.443 | 2.077 | 9.259 | 14.794 | 9.165 | 4.871 | 1.297 | 7.56 | 12.528 | -21.79 | 11.527 | 6.142 | 1.319 | 16.143 |
| 3 | -22.478 | 2.051 | 9.438 | 14.672 | 9.181 | 5.037 | 1.266 | 7.285 | 12.177 | -21.787 | 11.788 | 6.214 | 1.289 | 15.93 |
| Mean | -22.488 | 2.058 | 9.322 | 14.755 | 9.167 | 4.95 | 1.275 | 7.481 | 12.358 | -21.779 | 11.609 | 6.169 | 1.302 | 16.034 |
| BF | -1.567 |  |  |  |  |  |  |  |  |  |  |  |  |  |

Gelman-Rubin MCMC chain diagnostic test

|  | Point est. | Upper C.I. |
| --- | --- | --- |
| Dependent | 1 | 1.001 |
| Independent | 1 | 1.000 |

Table S70: Uto : Hawaiian <-> Bi.localextendedfamily

| run | MLL | q12 | q13 | q21 | q24 | q31 | q34 | q42 | q43 | MLL | α1 | β1 | α2 | β2 |
| --- | --- | --- | --- | --- | --- | --- | --- | --- | --- | --- | --- | --- | --- | --- |
| 1 | -30.775 | 8.311 | 9.53 | 10.87 | 11.001 | 4.341 | 5.867 | 9.315 | 13.714 | -30.63 | 12.774 | 7.016 | 7.521 | 14.356 |
| 2 | -30.651 | 8.501 | 9.562 | 11.08 | 11.166 | 4.404 | 5.834 | 9.123 | 13.545 | -30.64 | 12.927 | 7.046 | 7.587 | 14.498 |
| 3 | -30.579 | 8.401 | 9.297 | 10.802 | 11.189 | 4.185 | 5.732 | 8.967 | 13.385 | -30.67 | 12.829 | 7.013 | 7.597 | 14.707 |
| Mean | -30.668 | 8.404 | 9.463 | 10.917 | 11.119 | 4.31 | 5.811 | 9.135 | 13.548 | -30.65 | 12.843 | 7.025 | 7.568 | 14.52 |
| BF | -0.298 |  |  |  |  |  |  |  |  |  |  |  |  |  |

Gelman-Rubin MCMC chain diagnostic test

|  | Point est. | Upper C.I. |
| --- | --- | --- |
| Dependent | 1 | 1 |
| Independent | 1 | 1 |

Table S71: Uto : Hawaiian <-> Bi.localresidence

| run | MLL | q12 | q13 | q21 | q24 | q31 | q34 | q42 | q43 | MLL | α1 | β1 | α2 | β2 |
| --- | --- | --- | --- | --- | --- | --- | --- | --- | --- | --- | --- | --- | --- | --- |
| 1 | -26.28 | 1.668 | 11.853 | 13.363 | 10.441 | 7.761 | 5.103 | 3.591 | 16.547 | -26.20 | 12.714 | 6.925 | 3.791 | 18.639 |
| 2 | -26.36 | 1.651 | 11.773 | 13.286 | 10.512 | 7.803 | 5.12 | 3.708 | 16.532 | -26.13 | 12.713 | 6.921 | 3.755 | 18.874 |
| 3 | -26.44 | 1.744 | 11.523 | 13.638 | 10.217 | 7.639 | 4.985 | 3.719 | 16.345 | -26.16 | 12.578 | 6.891 | 3.777 | 18.75 |
| Mean | 26.36 | 1.688 | 11.716 | 13.429 | 10.39 | 7.734 | 5.069 | 3.673 | 16.475 | -26.16 | 12.668 | 6.912 | 3.774 | 18.754 |
| BF | -0.171 |  |  |  |  |  |  |  |  |  |  |  |  |  |

Gelman-Rubin MCMC chain diagnostic test

|  | Point est. | Upper C.I. |
| --- | --- | --- |
| Dependent | 1 | 1 |
| Independent | 1 | 1 |

Table S72: Uto : Iroquois <-> Cross.cousinmarriage.permitted

| run | MLL | q12 | q13 | q21 | q24 | q31 | q34 | q42 | q43 | MLL | α1 | β1 | α2 | β2 |
| --- | --- | --- | --- | --- | --- | --- | --- | --- | --- | --- | --- | --- | --- | --- |
| 1 | -19.063 | 1.874 | 6.128 | 18.046 | 4.835 | 19.191 | 2.455 | 9.444 | 10.638 | -18.30 | 6.008 | 18.784 | 1.774 | 18.58 |
| 2 | -19.181 | 1.803 | 6.142 | 17.771 | 4.938 | 19.232 | 2.38 | 9.378 | 10.8 | -18.33 | 6.029 | 18.792 | 1.775 | 18.544 |
| 3 | -19.079 | 1.844 | 6.049 | 17.919 | 4.941 | 18.973 | 2.421 | 9.401 | 10.485 | -18.25 | 6.017 | 18.811 | 1.778 | 18.477 |
| Mean | -19.108 | 1.84 | 6.106 | 17.912 | 4.905 | 19.132 | 2.419 | 9.408 | 10.641 | -18.28 | 6.018 | 18.796 | 1.776 | 18.534 |
| BF | -1.607 |  |  |  |  |  |  |  |  |  |  |  |  |  |

Gelman-Rubin MCMC chain diagnostic test

|  | Point est. | Upper C.I. |
| --- | --- | --- |
| Dependent | 1 | 1 |
| Independent | 1 | 1 |

Table S73: Uto : Iroquois <-> Cross.cousinmarriage.preferred

| run | MLL | q12 | q13 | q21 | q24 | q31 | q34 | q42 | q43 | MLL | α1 | β1 | α2 | β2 |
| --- | --- | --- | --- | --- | --- | --- | --- | --- | --- | --- | --- | --- | --- | --- |
| 1 | -21.001 | 2.198 | 6.215 | 9.527 | 4.62 | 18.329 | 2.978 | 11.337 | 12.544 | -20.41 | 6.098 | 18.901 | 2.313 | 12.189 |
| 2 | -21.062 | 2.199 | 6.212 | 9.481 | 4.489 | 18.401 | 2.981 | 11.133 | 12.646 | -20.48 | 6.016 | 18.785 | 2.346 | 12.303 |
| 3 | -21.042 | 2.164 | 6.214 | 9.399 | 4.54 | 18.54 | 2.98 | 11.208 | 12.415 | -20.44 | 6.038 | 18.734 | 2.332 | 11.955 |
| Mean | -21.035 | 2.187 | 6.214 | 9.469 | 4.55 | 18.423 | 2.98 | 11.226 | 12.535 | -20.44 | 6.051 | 18.807 | 2.33 | 12.149 |
| BF | -1.189 |  |  |  |  |  |  |  |  |  |  |  |  |  |

Gelman-Rubin MCMC chain diagnostic test

|  | Point est. | Upper C.I. |
| --- | --- | --- |
| Dependent | 1 | 1 |
| Independent | 1 | 1 |

Table S74: Uto : Iroquois <-> Exogamy.unilineal.descent

| run | MLL | q12 | q13 | q21 | q24 | q31 | q34 | q42 | q43 | MLL | α1 | β1 | α2 | β2 |
| --- | --- | --- | --- | --- | --- | --- | --- | --- | --- | --- | --- | --- | --- | --- |
| 1 | -19.857 | 1.932 | 5.193 | 18.951 | 5.548 | 16.727 | 2.342 | 8.695 | 10.838 | -19.20 | 5.327 | 16.263 | 1.785 | 19.204 |
| 2 | -19.857 | 1.97 | 5.198 | 19.098 | 5.449 | 16.791 | 2.344 | 8.594 | 11.127 | -19.09 | 5.349 | 16.39 | 1.775 | 19.377 |
| 3 | -19.869 | 1.956 | 5.278 | 19.102 | 5.306 | 16.811 | 2.363 | 8.67 | 11.019 | -19.14 | 5.398 | 16.558 | 1.769 | 19.544 |
| Mean | -19.861 | 1.953 | 5.223 | 19.05 | 5.434 | 16.776 | 2.35 | 8.653 | 10.995 | -19.14 | 5.358 | 16.404 | 1.776 | 19.375 |
| BF | -1.319 |  |  |  |  |  |  |  |  |  |  |  |  |  |

Gelman-Rubin MCMC chain diagnostic test

|  | Point est. | Upper C.I. |
| --- | --- | --- |
| Dependent | 1 | 1 |
| Independent | 1 | 1 |

Table S75: Uto : Iroquois <-> High.polygyny

| run | MLL | q12 | q13 | q21 | q24 | q31 | q34 | q42 | q43 | MLL | α1 | β1 | α2 | β2 |
| --- | --- | --- | --- | --- | --- | --- | --- | --- | --- | --- | --- | --- | --- | --- |
| 1 | -23.396 | 2.502 | 2.665 | 15.353 | 9.714 | 15.527 | 6.503 | 9.305 | 7.858 | -24.323 | 4.802 | 17.655 | 3.476 | 14.837 |
| 2 | -23.429 | 2.602 | 2.668 | 15.99 | 9.751 | 15.819 | 6.594 | 9.478 | 7.882 | -24.25 | 4.813 | 17.715 | 3.491 | 14.798 |
| 3 | -23.556 | 2.538 | 2.711 | 15.703 | 9.558 | 15.598 | 6.439 | 9.431 | 7.899 | -24.22 | 4.716 | 17.393 | 3.481 | 14.692 |
| Mean | -23.46 | 2.547 | 2.681 | 15.682 | 9.674 | 15.648 | 6.512 | 9.405 | 7.88 | -24.264 | 4.777 | 17.588 | 3.483 | 14.776 |
| BF | 1.854 |  |  |  |  |  |  |  |  |  |  |  |  |  |

Gelman-Rubin MCMC chain diagnostic test

|  | Point est. | Upper C.I. |
| --- | --- | --- |
| Dependent | 1 | 1.001 |
| Independent | 1 | 1.000 |

Table S76: Uto : Iroquois <-> Matri.anvunclocalresidence

| run | MLL | q12 | q13 | q21 | q24 | q31 | q34 | q42 | q43 | MLL | α1 | β1 | α2 | β2 |
| --- | --- | --- | --- | --- | --- | --- | --- | --- | --- | --- | --- | --- | --- | --- |
| 1 | -23.74 | 3.709 | 5.2 | 15.888 | 3.246 | 16.871 | 2.763 | 11.271 | 11.102 | -23.11 | 4.764 | 17.368 | 3.48 | 16.716 |
| 2 | -23.90 | 3.71 | 5.098 | 15.869 | 3.21 | 16.964 | 2.726 | 11.176 | 11.169 | -23.18 | 4.718 | 17.329 | 3.385 | 16.708 |
| 3 | -23.77 | 3.705 | 5.025 | 16.002 | 3.288 | 16.726 | 2.778 | 11.181 | 11.147 | -23.20 | 4.748 | 17.52 | 3.417 | 16.697 |
| Mean | -23.80 | 3.708 | 5.108 | 15.92 | 3.248 | 16.854 | 2.756 | 11.209 | 11.139 | -23.16 | 4.743 | 17.406 | 3.427 | 16.707 |
| BF | -1.269 |  |  |  |  |  |  |  |  |  |  |  |  |  |

Gelman-Rubin MCMC chain diagnostic test

|  | Point est. | Upper C.I. |
| --- | --- | --- |
| Dependent | 1 | 1 |
| Independent | 1 | 1 |

Table S77: Uto : Iroquois <-> Uni.linealdescent

| run | MLL | q12 | q13 | q21 | q24 | q31 | q34 | q42 | q43 | MLL | α1 | β1 | α2 | β2 |
| --- | --- | --- | --- | --- | --- | --- | --- | --- | --- | --- | --- | --- | --- | --- |
| 1 | -21.56 | 2.016 | 1.526 | 13.847 | 12.606 | 15.418 | 14.468 | 7.107 | 4.671 | -26.41 | 4.466 | 17.233 | 4.945 | 14.777 |
| 2 | -21.74 | 1.999 | 1.571 | 13.764 | 12.671 | 15.368 | 14.612 | 7.226 | 4.677 | -26.42 | 4.412 | 17.08 | 4.886 | 14.487 |
| 3 | -21.82 | 2 | 1.549 | 13.637 | 13.198 | 15.486 | 14.877 | 7.239 | 4.588 | -26.46 | 4.447 | 17.223 | 4.862 | 14.682 |
| Mean | -21.71 | 2.005 | 1.549 | 13.749 | 12.825 | 15.424 | 14.652 | 7.191 | 4.645 | -26.43 | 4.442 | 17.179 | 4.898 | 14.649 |
| BF | 9.705 |  |  |  |  |  |  |  |  |  |  |  |  |  |

Gelman-Rubin MCMC chain diagnostic test

|  | Point est. | Upper C.I. |
| --- | --- | --- |
| Dependent | 1.001 | 1.002 |
| Independent | 1.000 | 1.000 |

Table S78: Uto : Iroquois <-> Uni.localresidence

| run | MLL | q12 | q13 | q21 | q24 | q31 | q34 | q42 | q43 | MLL | α1 | β1 | α2 | β2 |
| --- | --- | --- | --- | --- | --- | --- | --- | --- | --- | --- | --- | --- | --- | --- |
| 1 | -25.58 | 5.895 | 1.555 | 19.537 | 10.054 | 16.03 | 13.63 | 9.314 | 4.858 | -28.22 | 4.721 | 17.299 | 9.254 | 17.468 |
| 2 | -25.69 | 5.995 | 1.607 | 19.745 | 9.926 | 16.301 | 13.722 | 9.422 | 4.883 | -28.30 | 4.678 | 17.135 | 9.263 | 17.446 |
| 3 | -25.61 | 6.066 | 1.56 | 20.025 | 9.889 | 16.235 | 13.52 | 9.286 | 4.752 | -28.26 | 4.812 | 17.359 | 9.356 | 17.475 |
| Mean | -25.63 | 5.985 | 1.574 | 19.769 | 9.956 | 16.189 | 13.624 | 9.341 | 4.831 | -28.26 | 4.737 | 17.264 | 9.291 | 17.463 |
| BF | 5.29 |  |  |  |  |  |  |  |  |  |  |  |  |  |

Gelman-Rubin MCMC chain diagnostic test

|  | Point est. | Upper C.I. |
| --- | --- | --- |
| Dependent | 1 | 1.000 |
| Independent | 1 | 1.001 |

###

### Bantu node 70 state inference

Here we re-construct node 70 within the Bantu tree, to confirm the hypotheses that the presence of both Iroquois terminologies and a preference for cross-cousin marriage existed at this point.

Table 1 in the main text shows that the dependent model is already preferred, so here we only look at the dependent model. The figure below shows the likelihood of each possibility. Top left: Absence of both Iroquois terminology and a preference for cross-cousin marriage. Top right: Absence of cross-cousin marriage and the presence of cross-cousin marriage preference. Bottom right: Both Iroquoian terminologies and cross-cousin marriage preferences are present. Bottom left: presence of an Iroquoian terminology, and absence of a cross-cousin marriage preference.

The graph clearly shows the presence of both traits being present here is the most likely option, with approximately 0.77 probability. This probability drops to around 0.5 on the ancestral node.


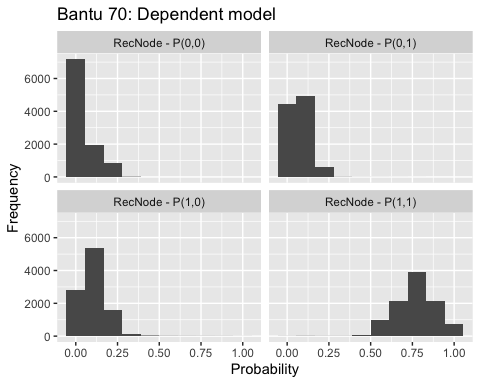


## Multiple comparisons

Since this study has numerous hypothesis tests, it is important we address the statistical problem of multiple comparisons. The multiple comparison problem says that the more hypotheses tested simultaneously, the more likely an erroneous error is to occur. There is strong debate in the literature on whether this is a problem here, but we argue that it is not.

The primary reason for this not being a problem is that all our hypotheses were set out *a priori*. They are not the result of comparing all social variables against all binary kinship terminologies and make up a tiny portion of those that are possible. All hypotheses are established in theory and many had been previously tested.

## Phylogenetic Inertia

We were concerned that a prevalence of phylogenetic inertia was the primary reason for not finding many significant co-evolutionary relationships. That is, kinship terminologies and social organisation are so tightly tied, that we would not be able to detect a relationship using this approach. To assess how prevalent this was in our results we reviewed each hypothesis on 3 criteria; 1) What was the most likely independent ancestral state, 2) How likely was that ancestral state, and 3) How are the two variables distributed across the respective phylogenies. In an independent model (where each trait changes independent of the other) we would expect that if the ancestral state shows the presence of both traits, there is a possibility that both traits were inherited over time, either by chance or due to a stable relationship. In both these cases, our phylogenetic approach would fail to pick up a relationship between these traits. The second criteria give us a level of confidence in the first criteria, we assume a probability of above 0.7 indicates a high likelihood of the true ancestral state. The third criteria allow us to qualitatively review whether we think the methods are failing to detect a relationship, by visualizing the data on mirrored phylogenies. The first two criteria mean that it is plausible that two traits are ancestral, but unrelated, by looking at the distribution of traits on a tree, we can assess whether this is the case or not. Visualization also allows us to attest whether stable pairings occur later within a lineage. These plots can be found in the figures folder in the GitHub repository. Using the three criteria above, we then decide if a hypothesis is likely to be influenced by phylogenetic inertia on a four-point scale, ranging from highly likely to highly unlikely. The table below shows these judgments, and ancestral state criteria, for all hypotheses that we did not find support for. These decisions were independently decided on by each author and then conferred on the result.

Table S85: Judgments of phylogenetic inertia. The first columns show our overall judgements. The second column shows the most likely ancestral state in an independent model of evolution, organised as [Kin terminology], [social structure]. The third column shows the probability of this state.

| hypothesis | Most likely ancestral state | Probability of ancestral state | judgement |
| --- | --- | --- | --- |
| austronesian-crow-high.polygyny | 0,0 | 0.99 | Highly unlikely |
| austronesian-crow-polygyny | 0,1 | 0.64 | Unlikely |
| austronesian-eskimo-absenceofcousinmarriage.permitted | 1,1 | 0.42 | Unlikely |
| austronesian-eskimo-absenceofcousinmarriage.preference | 1,0 | 0.81 | Highly unlikely |
| austronesian-eskimo-bi.linealdescent | 1,0 | 0.91 | Highly unlikely |
| austronesian-eskimo-neo.localresidence | 1,0 | 0.79 | Highly unlikely |
| austronesian-eskimo-nuclear.families | 1,1 | 0.74 | **Likely** |
| austronesian-hawaiian-absenceofcousinmarriage.permitted | 1,1 | 0.43 | Unlikely |
| austronesian-hawaiian-absenceofcousinmarriage.preference | 1,0 | 0.52 | Unlikely |
| austronesian-hawaiian-bi.linealdescent | 0,0 | 0.44 | Unlikely |
| austronesian-hawaiian-bi.localextendedfamily | 1,1 | 0.3 | Unlikely |
| austronesian-hawaiian-bi.localresidence | 1,0 | 0.5 | Highly unlikely |
| austronesian-iroquois-exogamy.unilineal.descent | 0,0 | 0.99 | Highly unlikely |
| austronesian-iroquois-high.polygyny | 0,0 | 0.99 | Highly unlikely |
| austronesian-iroquois-matri.anvunclocalresidence | 0,0 | 0.96 | Highly unlikely |
| austronesian-iroquois-polygyny | 0,1 | 0.62 | Highly unlikely |
| austronesian-iroquois-uni.linealdescent | 0,0 | 0.92 | Highly unlikely |
| bantu-crow-matri.anvunclocalresidence | 0,0 | 0.65 | Highly unlikely |
| bantu-crow-matrilineal | 0,0 | 0.68 | Highly unlikely |
| bantu-crow-matrilocal | 0,0 | 0.97 | Highly unlikely |
| bantu-crow-uni.localresidence | 0,1 | 0.95 | Highly unlikely |
| bantu-hawaiian-absenceofcousinmarriage.permitted | 0,1 | 0.879 | Highly unlikely |
| bantu-hawaiian-absenceofcousinmarriage.preference | 0,0 | 0.9 | Highly unlikely |
| bantu-hawaiian-bi.linealdescent | 0,0 | 0.89 | Highly unlikely |
| bantu-hawaiian-bi.localextendedfamily | 0,0 | 0.73 | Highly unlikely |
| bantu-hawaiian-bi.localresidence | 0,0 | 0.92 | Highly unlikely |
| bantu-iroquois-cross.cousinmarriage.preferred | 1,1 | 0.51 | Highly likely |
| bantu-iroquois-exogamy.unilineal.descent | 0,0 | 0.44 | Highly unlikely |
| bantu-iroquois-uni.linealdescent | 1,1 | 0.59 | Unlikely |
| bantu-iroquois-uni.localresidence | 1,1 | 0.5 | **Likely** |
| bantu-omaha-uni.localresidence | 0,1 | 0.93 | Highly unlikely |
| uto-hawaiian-absenceofcousinmarriage.permitted | 1,1 | 0.98 | Unlikely |
| uto-hawaiian-absenceofcousinmarriage.preference | 1,0 | 0.99 | **Likely** |
| uto-hawaiian-bi.linealdescent | 1,0 | 0.98 | Highly unlikely |
| uto-hawaiian-bi.localextendedfamily | 1,0 | 0.74 | Unlikely |
| uto-hawaiian-bi.localresidence | 1,0 | 0.98 | Unlikely |
| uto-iroquois-cross.cousinmarriage.permitted | 0,0 | 0.99 | Highly unlikely |
| uto-iroquois-cross.cousinmarriage.preferred | 0,0 | 0.99 | Highly unlikely |
| uto-iroquois-exogamy.unilineal.descent | 0,0 | 0.99 | Highly unlikely |
| uto-iroquois-high.polygyny | 0,0 | 0.99 | Highly unlikely |
| uto-iroquois-matri.anvunclocalresidence | 0,0 | 0.95 | Highly unlikely |
| uto-iroquois-uni.linealdescent | 0,0 | 0.99 | Highly unlikely |
| uto-iroquois-uni.localresidence | 0,0 | 0.49 | Highly unlikely |

# References

Currie, T. E., Greenhill, S. J., Gray, R. D., Hasegawa, T., & Mace, R. (2010). Rise and fall of political complexity in island South-East Asia and the Pacific. *Nature*, *467*(7317), 801–804. https://doi.org/10.1038/nature09461

Dunn, M., Greenhill, S. J., Levinson, S. C., & Gray, R. D. (2011). Evolved structure of language shows lineage-specific trends in word-order universals. *Nature*, *473*(7345), 79–82. https://doi.org/10.1038/nature09923

Gelman, A., & Rubin, D. B. (1992). Inference from Iterative Simulation Using Multiple Sequences. *Statistical Science*, *7*(4), 457–472. https://doi.org/10.1214/ss/1177011136

Gray, R. D., Drummond, A. J., & Greenhill, S. J. (2009). Language Phylogenies Reveal Expansion Pulses and Pauses in Pacific Settlement. *Science*, *323*(5913), 479–483. https://doi.org/10.1126/science.1166858

Grollemund, R., Branford, S., Bostoen, K., Meade, A., Venditti, C., & Pagel, M. (2015). Bantu expansion shows that habitat alters the route and pace of human dispersals. *Proceedings of the National Academy of Sciences*, *112*(43), 13296–13301. https://doi.org/10.1073/pnas.1503793112

Kirby, K. R., Gray, R. D., Greenhill, S. J., Jordan, F. M., Gomes-Ng, S., Bibiko, H.-J., Blasi, D. E., Botero, C. A., Bowern, C., Ember, C. R., Leehr, D., Low, B. S., McCarter, J., Divale, W., & Gavin, M. C. (2016). D-PLACE: A Global Database of Cultural, Linguistic and Environmental Diversity. *PLOS ONE*, *11*(7), e0158391. https://doi.org/10.1371/journal.pone.0158391

Pagel, M., & Meade, A. (2017). Bayes Traits V3. *Reading: University of Reading*.

Pagel, Mark, & Meade, A. (2006). Bayesian Analysis of Correlated Evolution of Discrete Characters by Reversible‐Jump Markov Chain Monte Carlo. *The American Naturalist*, *167*(6), 808–825. https://doi.org/10.1086/503444
